# Flat-panel electronic displays: a triumph of physics, chemistry and engineering

**DOI:** 10.1098/rsta.2009.0247

**Published:** 2010-03-13

**Authors:** Cyril Hilsum

**Affiliations:** University College London, London, UK

**Keywords:** displays, cathode ray tube, liquid crystals

## Abstract

This paper describes the history and science behind the development of modern flat-panel displays, and assesses future trends. Electronic displays are an important feature of modern life. For many years the cathode ray tube, an engineering marvel, was universal, but its shape was cumbersome and its operating voltage too high. The need for a flat-panel display, working at a low voltage, became imperative, and much research has been applied to this need. Any versatile flat-panel display will exploit an electro-optical effect, a transparent conductor and an addressing system to deliver data locally. The first need is to convert an electrical signal into a visible change. Two methods are available, the first giving emission of light, the second modulating ambient illumination. The most useful light-emitting media are semiconductors, historically exploiting III–V or II–VI compounds, but more recently organic or polymer semiconductors. Another possible effect uses gas plasma discharges. The modulating, or subtractive, effects that have been studied include liquid crystals, electrophoresis, electrowetting and electrochromism.

A transparent conductor makes it possible to apply a voltage to an extended area while observing the results. The design is a compromise, since the free electrons that carry current also absorb light. The first materials used were metals, but some semiconductors, when heavily doped, give a better balance, with high transmission for a low resistance. Delivering data unambiguously to a million or so picture elements across the display area is no easy task. The preferred solution is an amorphous silicon thin-film transistor deposited at each cross-point in an *X*–*Y* matrix.

Success in these endeavours has led to many applications for flat-panel displays, including television, flexible displays, electronic paper, electronic books and advertising signs.

## 1. Background

Electronic displays are an important feature of modern life. They have a magical allure, for they make complex operations within electronic circuits visible. The father of all displays, the cathode ray tube (CRT), was invented at more or less the same time as the vacuum valve, and as the valve improved in performance, and as circuits grew more complex, so visualizing the outputs became essential, and here the CRT was supreme. The invention of television (TV) made more demands on the output device, and the CRT developed into an engineering marvel, giving a multi-colour representation of any scene, still or moving, that matched the original in fine detail. Moreover, this device was made in such numbers and at such a low cost that it could be found in most homes in the developed world.

However, the design of the CRT gave some serious problems. The first was the problem of making big screens. It is difficult to bend electrons through wide angles, so a large-area display had to incorporate a long tube for the electron source. Further, because thick glass is needed for the face plate, in order to withstand the air pressure, large tubes are very heavy, a set with a 36 inch diagonal screen weighing over 100 kg. Other negative features are the need for high-voltage operation to energize the phosphors, the risk of X-rays, and the life limit of perhaps 5 years. Scientists were conscious of these limitations and there were many attempts to invent simpler forms of CRT. Particularly noteworthy were the tubes proposed by Ross Aiken and by Denes (later Dennis) Gabor, with the electron gun at the side of the screen. These gave the system a smaller volume, but could not avoid the other disadvantages.

**Figure 1.**
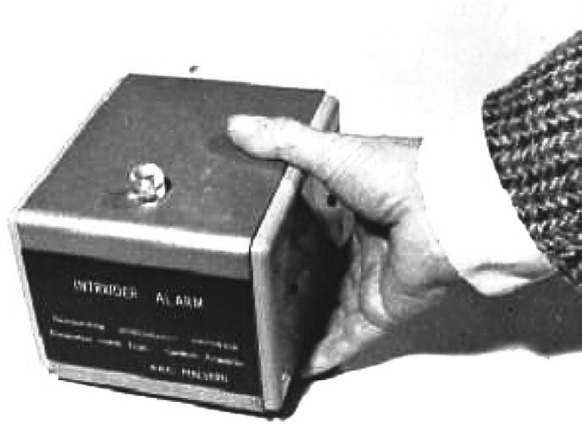
A mini-radar set.

At this time, the 1950s and 1960s, electronics was experiencing a revolution. The invention of the transistor had been followed by the integrated circuit, with several thousand devices packed into a square centimetre, and all driven by a few volts. The CRT was becoming more and more exposed as a dinosaur. The major potential return from the invention of a display that matched the virtues of the new electronics induced many companies to undertake research in the field. Defence laboratories, already anxious to exact real benefits from the simplicity and reliability of integrated circuits, were also conscious of the need to find a solid-state display. A simple Doppler radar set could work off a few volts, and could be held in the hand (figure 1); while the electronic display to show the output needed a high voltage and much more space.

## 2. Display requirements

There are three basic requirements for any flat-panel display capable of showing a high-resolution colour picture. They are:

— an electro-optical effect;
— a transparent conductor;
— a nonlinear addressing component.

The first need is to convert an electrical signal into a visible change. There are a large number of potential candidates for this, and they fall into two classes. The first gives emission of light; the second modulates ambient illumination, or light from an artificial source, generally behind the display panel.

The most useful light-emitting media are semiconductors, historically exploiting III–V or II–VI compounds, but now, more successfully, organic or polymer semiconductors. There are persistent attempts to breathe new life into the flat CRT, and much effort into maintaining interest in gas discharge tubes. Modulating, or subtractive, effects are legion, and the literature abounds with new candidates, or revisions of old failed ones. Those that have shown success or potential include liquid crystals, electrophoresis, electrowetting and electrochromism, in both solids and liquids. Effects that have not survived include dichroism, whereby elongated crystals with anisotropic absorption are persuaded to move in an electric field—crossed crystals absorb, parallel crystals transmit. The favoured medium was herapathite, quinine iodine sulphate, used in the first sheet polarizers. This was named after a Bristol doctor, William Herapath, who observed that when one of his students, a Mr Phelps, added iodine to the urine of a dog that had been fed quinine, green crystals grew in the ‘reaction liquid’ (Land 1951). History does not record the later career of such an imaginative student, but, alas, it does record the failure of dichroic displays.

The need for a transparent conductor is obvious, since one needs to apply a voltage to an extended area while observing the results. The current is carried by the free electrons, but these also absorb incident radiation. There is then a balance in the design, because, as the thickness of the coating is increased to lower the resistance, so the transmission decreases. The first materials used were metals, and the top electrode in early selenium photocells was a very thin gold film. For all metals, the absorption is so high that the electrode can have a thickness of only a few hundred ångströms, and it is then difficult to deposit a film of uniform thickness over a large area. Some semiconductors, when heavily doped, give a better balance, with high transmission for a low resistance.

**Figure 2.**
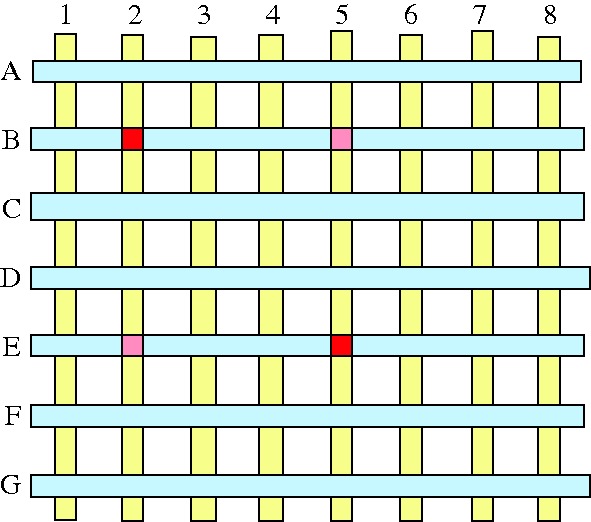
Matrix addressing.

Delivering picture or symbol data to the display is no easy task. Each picture element, or pixel, must be addressed individually, without crosstalk, and a large-area TV screen can have well over a million pixels. It would be impracticable to have two connectors to each pixel, and a matrix of orthogonal *X* and *Y* lines is always used, with the cross-points labelled as an address. The technique is called matrix addressing (figure 2). Applying half the required voltage to, say, column 2 and row B would certainly excite solely pixel 2:B, but the simultaneous excitation of pixel 5:E would also switch on pixels 2:E and 5:B.

**Figure 3.**
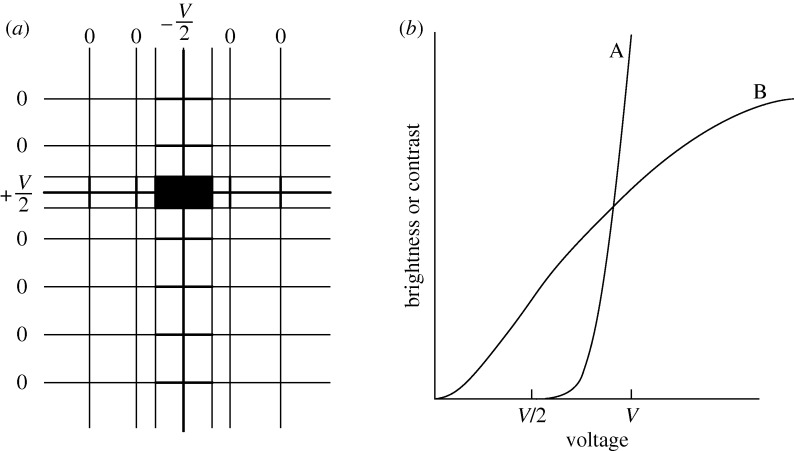
Matrix addressing problems. (*a*) Multiplexing. (*b*) Electro-optical effects with (curve A) and without (curve B) a threshold.

**Figure 4.**
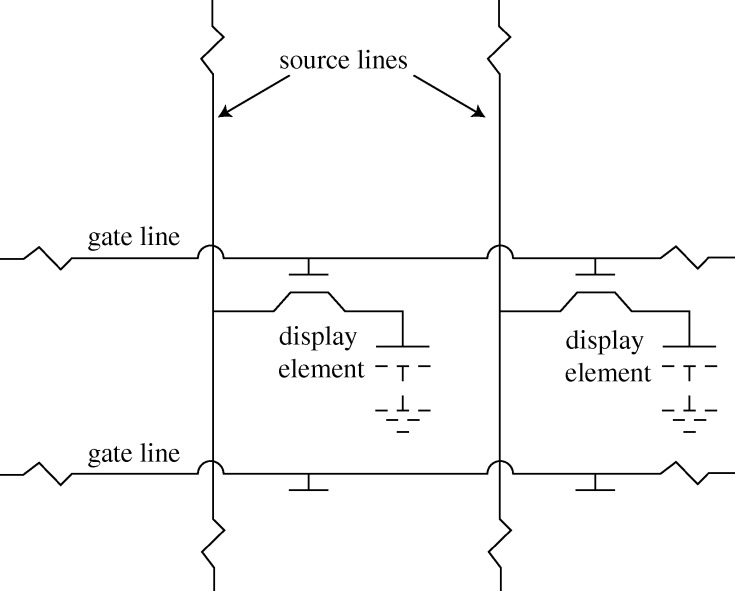
The active matrix.

The remedy is sequential activation, or multiplexing, running down the *X* rows one by one, applying the half-voltage to the appropriate *Y* columns at the correct time. While this does solve the ambiguity problem, it creates a crosstalk problem, in that a number of half-voltages will be applied to pixels that should have been passive. Figure 3*a* shows that, whenever a pixel is addressed, there is a possibility of a cross-bar pattern emerging. The situation would be tolerable for a small display exploiting an electro-optical effect with a voltage threshold, as in curve A (figure 3*b*), where a half-voltage impulse has no effect at all, but most useful effects have an inadequately sharp threshold, showing a fairly gradual change in transmission with voltage, as in curve B. The solution is to add to each pixel a circuit element that does have a sharp threshold, such as a semiconductor diode or a transistor, converting the passive matrix into an active matrix (figure 4). The element must, of course, be integrated on the panel, in the form of a thin film. Early panels used CdS or CdSe thin-film transistors (TFTs), but these proved unstable and short-lived. Progress on flat-panel displays burgeoned only when amorphous silicon TFTs became available.

## 3. Transparent conductors

The display medium needs two electrodes, and at least one of these must be transparent, so that the optical path from the pixel to the eye will suffer little absorption. For most practical effects, the pixel electrodes are orthogonal to this path, and run the full width of the display. The conductivity of the electrodes must then be high enough to give a uniform voltage over the display area.

Let us assume that we will use a semiconductor for the electrode. Drude theory shows that free carrier absorption will give an absorption coefficient *α* at a wavelength λ as
3.1
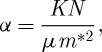

where *N* is the electron concentration, *μ* is the mobility, m* is the electron effective mass and

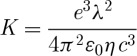

with *η* the refractive index. A numerical factor should also be included to allow for averaging over the electron energies, and this factor will depend on the relative contributions of scattering by the lattice, impurities, surfaces and grain boundaries.

For a film of thickness *t* cm with absorption *A* less than about 20 per cent,
3.2
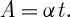



The resistance per square (*R*_s_) is given by
3.3
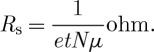

The electrode must be colourless, so the semiconductor energy gap will be greater than 3 eV. The electron effective mass m* will then be greater than 0.1*m*_0_, so the mobility *μ* will certainly be less than 200 cm^2^ V^−1^ s^−1^. An increase in *N* will reduce *R*_s_, though there will be an increase in impurity scattering, so that the mobility will fall. Typically we require *R*_s_ to be between 1 and 100 Ω, depending on the type and area of the display, and a convenient film thickness will be between 0.1 and 10 μm, so we can aim at *N* approaching 10^21^ cm^−3^ combined with mobilities around 30 cm^2^ V^−1^ s^−1^. However, the absorption increases with *N*, so, since we need both conductivity and transparency, designing a transparent conductor is a matter of swings and roundabouts. One factor of merit is the product *AR*_s_, which is independent of carrier concentration and thickness, since
3.4
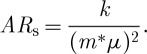

We wish to minimize this product. Substitution of typical values for the parameters involved in *k* shows that, with an effective mass ratio of 0.3 and a mobility of 30 cm^2^ V^−1^ s^−1^, *AR*_s_ is approximately 60. So it should be possible to obtain a resistance per square of 20 Ω with a film absorption of 3 per cent.

Some 30 years ago it was discovered that cadmium oxide had a low *AR*_s_ product, and it was widely used. It was in turn replaced by tin oxide, which then became a standard. In the 1970s indium oxide doped with tin (ITO), prepared by spraying, was shown to give better results still, but today layers are prepared by sputtering, a more practicable technique for displays. Such layers have been reported with *N* near 10^21^ cm^−3^ and a mobility of 43 cm^2^ V^−1^ s^−1^. Such a combination is remarkable in a material of such a large energy gap, particularly when we remember that we are dealing with a polycrystalline layer. *AR*_s_ products below 50 have been reported for ITO, but an alternative compound, cadmium stannate, has shown *AR*_s_ values a factor of 2 lower. Unfortunately, cadmium is highly toxic, and is rarely used in production.

This does not mean that ITO has no rivals. Though it has excellent parameters, there are some disadvantages. The high usage of such coatings has caused the price of indium to escalate, and there is even the possibility of a world shortage. A more definite disadvantage is the lack of flexibility in thick coatings, which makes ITO suspect as the electrode for flexible displays. A possible alternative is ZnO doped with Al. Sputtered layers have shown values for *AR*_s_ as low as those for ITO (Igasaki & Saito 1991*a*,*b*).

The transparent conducting electrode represents an important achievement for material science that is rarely publicized.

## 4. The visual effect

There is a clear distinction between emissive displays, which work by generating light, and subtractive displays, which exploit modulation of incident or transmitted light by controlling absorption, reflection or scattering. Both classes have their proponents, since each has shortcomings. Subtractive displays are economic in power, but in the absence of ambient light a subsidiary source is needed, and much of the power advantage is then lost. In ambient light, some subtractive effects can have a dull appearance, lacking charm. Emissive displays are attractive to view in low or moderate light levels, but they fade and are difficult to read when bright light falls on the panel. We still lack an efficient emissive technology, though there has been much progress in the past few years. To obtain sufficient brightness, it is necessary to drive the emitting pixels hard, and then their life can be curtailed.

### (a) Subtractive displays

Progress in semiconductor electronics after 1950 was so rapid that scientists assumed that semiconductors could do everything. Certainly our understanding of the physics of solids became deep and wide, and we could devise strategies for material design to meet a variety of applications. A number of electro-optical effects in solids were proposed for displays, but all fell short of the requirements, usually because they operated at high voltages. It was with some misgivings that the community realized that the solution could come from liquids, a previously neglected form of condensed matter, and one where much of our applied physics and engineering was inappropriate. To augment these concerns was the discovery that the best effects occurred in organic materials, previously the almost exclusive province of chemists. Some relief was felt when it was shown that families of candidate materials could be produced in weeks, in contrast to the years that were needed to make and purify inorganic semiconductors like silicon and gallium arsenide. The years when display concepts were being invented and refined were truly years of equal partnership between physicists and chemists.

The 1960s saw four rival subtractive technologies emerging, with little indication that any one had crucial advantages.

#### (i) Liquid crystals

**Figure 5.**
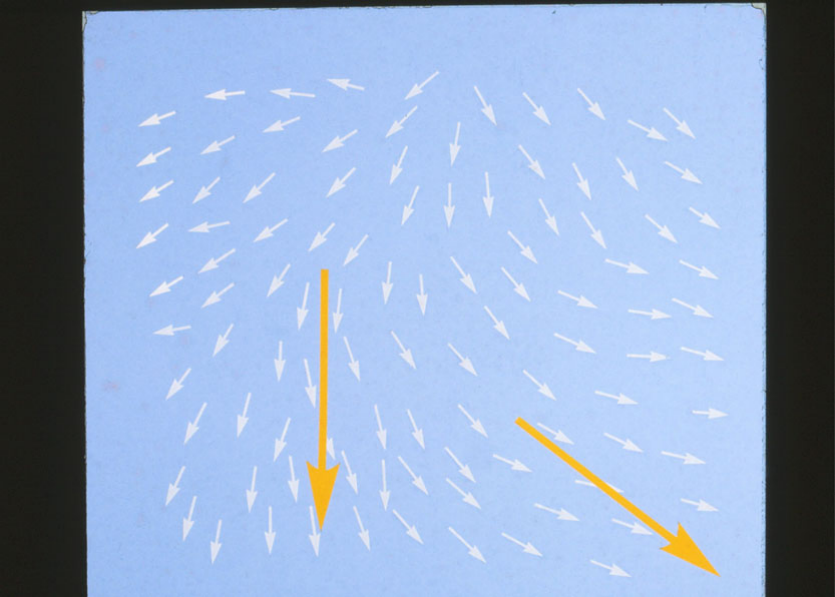
The director in a nematic liquid crystal.

Over 100 years ago an Austrian botanist, Reinitzer (1888), discovered a fourth state of matter, with properties intermediate between solids and classical liquids. This was a liquid that showed long-range order, though only over a limited temperature range. On melting from the frozen state, the long-range order made the liquid cloudy, but as the temperature was increased, it became clear. The molecules of the liquid were long and thin, and the long directions followed an ordered pattern. Further research revealed three types of order. Smectic crystals are similar in their order to crystalline solids, in that the molecules form equally spaced layers, all pointing in the same direction, though with little positional order within a layer. In nematic crystals, there are no layers, the molecules pointing in the same direction locally, though that direction, called the director, changes over a distance (figure 5). Cholesteric crystals are layered, but the molecules lie along the layers, all those in a layer pointing in the same direction, with that direction changing gradually and regularly with distance, so that the director follows a helix.

Liquid crystals (LCs) remained an academic curiosity until 1962, when Williams (1963), at the Radio Corporation of America’s (RCA) Sarnoff Research Center, discovered changes in the optical transmission of thin films of *para*-azoxyanisole held between two glass slides on the application of 12 V. Williams subsequently left the laboratory, but his lead was followed by George Heilmeier, who persuaded the RCA management to start an LC project. There was, at that time, no room-temperature LC, but the RCA chemists devised a mixture of three Schiff’s bases that had a nematic range from 22 to 105°C (Goldmacher & Castellano 1967). The effect that RCA wished to exploit in displays was called the dynamic scattering mode (DSM), in which the mixture turns from transparency to white opalescence over a range of a few volts (Heilmeier *et al*. 1968). LCs are anisotropic in almost all their physical characteristics. The values of refractive index, dielectric constant, conductivity, elasticity and viscosity are very different when measured along the long molecular axis or the short axes. Because of the dielectric anisotropy, the molecule will turn in an electric field, and nematics divide into two classes, positive crystals, which align along the field, and negative crystals, which try to lie across it. DSM can be generated in negative nematics, because charges build up along the molecules, giving rise to a field at right angles to the applied field. At higher fields, turbulence occurs. RCA publicized their discoveries in 1968, and, amid some excitement, many companies set about exploiting liquid crystal displays (LCD) in digital watches and calculators. Curiously, RCA was an exception.

RCA had little interest in small instruments. Their display involvement was in CRTs, and here their position was almost unique. Harold Law and Al Schroeder had invented the shadow-mask tube some 10 years earlier, and this was now being made in quantity at plants in Lancaster, Pennsylvania, and Harrison, New Jersey (Law 1950, 1976). The company had early adopted the principle of licensing their inventions, and shadow-mask tubes were now produced worldwide.^1^ Actually, this policy was so successful that the royalty income financed the whole of the Sarnoff Research Center. It was not surprising that the Development Laboratory did not share the conviction that LCDs were the way forward for TV displays. They were conscious of the problems of addressing, and unconvinced that the RCA invention of the TFT in 1962 (Weimer 1962*a*,*b*) would be the solution. In any case, they did not see the virtue in trying to replace their own world-leading display, and did not accept the moral, ‘If you don’t do it, someone else will’.

Looking back, it is obvious that RCA held most of the assets needed to forge and preserve world leadership in flat-panel displays, but they opted out. The management set a target of a 1200 pixel integrated display panel to be made early in 1968, but when no panel was made by the end of the year, the project was cancelled. In the next year they abandoned all work on LC TV, though some work on small displays continued until 1972.

It would not be an overstatement to say that US industry lost its way on displays in the 1970s. We have seen that the early running on LCs was made by RCA. That laboratory had not been in the forefront of discovery on transistors and chips, relying mainly on licensing from Bell Telephone Laboratories (BTL), but it had a proud record of innovation in vacuum tubes, culminating in the invention of the shadow-mask CRT. RCA led in the early research on TFTs and LCDs, but the belief that flat-panel displays were against their long-term interests led them to withdraw from the field in 1972. The other potential industrial leader, BTL, had stayed curiously aloof from the frenzied search for novel display technology, partly because of their increased involvement in semiconductor lasers for communications, but also because senior figures in their laboratory were unconvinced that new displays were required. They said (Gordon & Anderson 1973):

Prospects for new display technologies are clouded by the fact that there exists a device, the familiar CRT, that has long provided a versatile, elegant, functional, economical, and largely satisfactory solution.

In circumstances where industry was unwilling to lead in long-term research programmes, defence funding had previously filled the gap, and we have seen that this indeed happened in the UK. In the USA, however, the Department of Defense (DoD) was also unconvinced about flat panels. The opposition was led, strangely, by the scientist who had contributed much to the earlier enthusiasm at RCA for LCs, George Heilmeier. He had left RCA in 1970, and within two years was holding a senior post in the US DoD with responsibility for electronic device research contracts. He told the main US Displays Conference (Heilmeier 1973):

How many realistic scenarios are there in which we win because we have a flat-panel, matrix-addressed display in the cockpit? We must feed on existing technologies.

It was not surprising then that in the 1970s most of the important developments in this field came from Europe and Japan.

**Figure 6.**
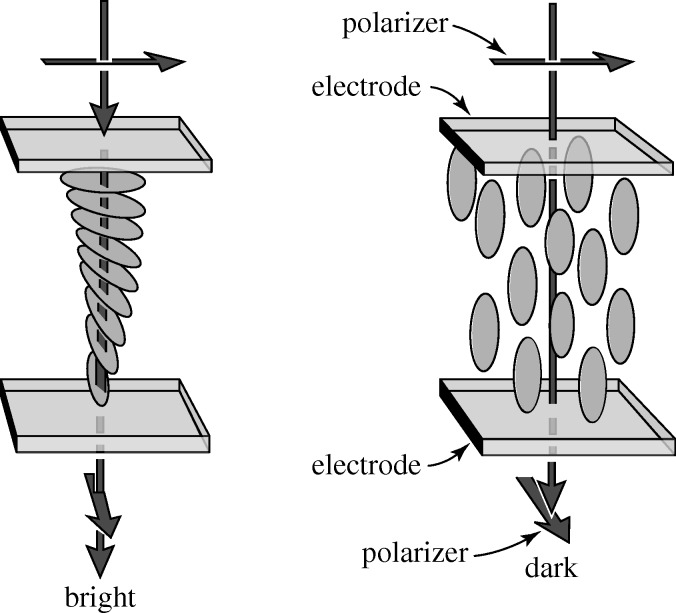
The twisted nematic display.

The lack of management interest in LCDs certainly led to a number of the RCA scientists leaving, and one of their best theorists, Wolfgang Helfrich, joined Hoffmann-La Roche (H-LR), the Swiss chemical and pharmaceutical company, in 1970. There he suggested to Martin Schadt, the LC group leader, that he should work on a new display effect that exploited positive nematics. Helfrich’s idea was to make a thin LC cell that rotated the plane of incident polarized light by 90°. It was known that nematic molecules would lie down on a glass substrate that had been rubbed with a polishing cloth in one direction. If that direction was orthogonal on the two surfaces, a 90° twist would be induced, and when the cell was put between parallel polarizers, no light could pass. However, if a field was applied across that cell, the molecules would align themselves along the field, the twist would disappear, and light could pass. Schadt made the cell, it worked, and the twisted nematic (TN) display was born (figure 6).

There were some curious features to this invention. Helfrich left RCA in October 1970, made the first TN cells in November, submitted a patent with Schadt on 4 December (Helfrich & Schadt 1970) and a letter to *Applied Physics Letters* 3 days later (Schadt & Helfrich 1971). Such a rapid sequence of conception and construction is unusual. In fact, as Helfrich admitted 20 years later, he had thought of the TN effect in 1969, and other ex-RCA staff confirmed this. However, he made little attempt to attract management interest, since, as he explained, he was there to help theoretical understanding, not to invent devices. RCA made no attempt to invalidate the patent or to claim ownership, possibly because there were further legal complications (Kawamoto 2002).

James Fergason was a scientist who had worked on cholesteric LCs at Westinghouse in the early 1960s, but left in 1966 to join Kent State University. Two years later he formed his own company, ILIXCO, to manufacture LC displays. In 1968 and 1970 he published two papers that effectively contained descriptions of the TN display (Arora *et al.* 1968; Fergason *et al*. 1970). He made no attempt then to patent the concept, and was surprised, and probably irritated, when a colleague reported back after a visit to H-LR that Schadt and Helfrich had invented a new form of LCD. In fact, it was as a result of this inadvertent disclosure that H-LR had rapidly taken the patenting and publishing actions. Fergason himself set about composing patents and, after an abortive attempt in February, submitted in April a patent, which was granted in 1973 (Fergason 1971). No mention was made in this patent of his earlier publications. Though the validity of Fergason’s patent could have been queried because of those disclosures, there could be no doubt that he had made and shown a device in April 1970, because he had recorded the invention in witnessed notebooks. He therefore had good grounds for contesting the H-LR patent, and after protracted legal proceedings this was withdrawn. However, H-LR regained legal ownership of TN rights by buying the Fergason patent from ILIXCO, which were in financial difficulties. A compromise agreement shared royalties amicably between all the interested parties except RCA.

Though the way was now legally clear for companies to exploit TN displays, the commercial position was unclear. A number of companies had taken licences from RCA to exploit dynamic scattering, and they were reluctant to adopt an untested technology. However, problems soon arose because of the LC material. DSM effects need negative nematics, and though RCA had now demonstrated a suitable Schiff’s base that was nematic at room temperature, it did not have an adequate working range. Sharp developed a eutectic mixture of three Schiff’s bases that worked over the range 0–40°C, but were then frustrated when their devices failed after only a few weeks of operation. It became apparent that there was no stable LC available, and LCDs were acquiring a poor reputation for reliability.

**Figure 7.**
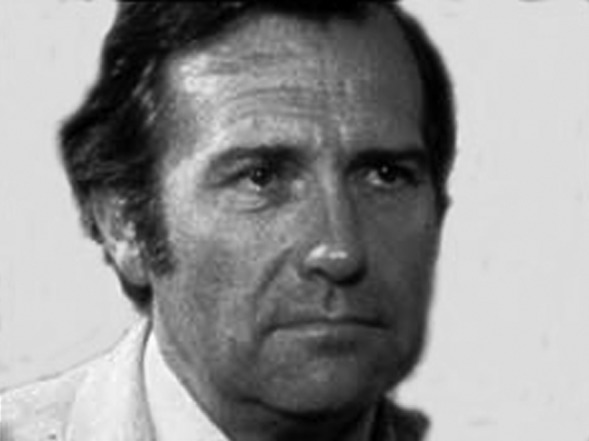
John Stonehouse, UK Minister of State for Technology, 1967–1968.

Up to then, the UK had played little part in LC development, though one or two university chemistry departments were involved in research, and one company, Marconi, had patented an LCD before the war (Levin & Levin 1934). Now events took a curious turn, because a politician became involved. Much UK semiconductor research had been carried out in government defence laboratories, and early development of LEDs and diode lasers had taken place at the Services Electronics Research Laboratory (SERL), Baldock, and at the Royal Radar Establishment (RRE), Malvern. One of the aims of the Labour Government elected in March 1966 had been to forge a ‘white-hot technological revolution’, and the next year they established a Ministry of Technology. This assimilated some of the defence laboratories, including RRE, and in March 1967 the Minister of State for Technology, John Stonehouse (figure 7), came to Malvern.^2^

He was surprised to hear that royalties to RCA on the shadow-mask tube cost the UK more than Concorde, and after overnight deliberation authorized the Director of RRE, Dr (later Sir) George Macfarlane, to start a programme on flat-panel electronic displays. Surprised at this rapid decision, and informed by senior staff that there was no expertise within RRE to mount a meaningful development programme, he set up a committee to study the field. This recommended in December 1969 that the UK Government should fund research on flat-panel electronic displays, with LCs as the first priority (Hilsum 1969).

Though formal approval of this recommendation would normally have taken some months, and, indeed, was never granted, RRE had anticipated approval, and justified their action on the urgent need for displays for the portable radar sets they had invented. They established two consortia, one for materials, involving RRE, Hull University and BDH, and one for devices, involving RRE, SERL, Marconi, Rank and STL. The Plessey Research Laboratory at Caswell were also involved, specializing in electrophoretics. Though most of these organizations were ‘the usual suspects’ from the semiconductor R&D programmes, Hull University were unknown. They had come to the attention of RRE during a meeting held to probe UK expertise on LCs, when it became clear that Hull, led by Professor George Gray, were clear leaders in the understanding of LC chemistry. This trust was rewarded manifold. Gray was given the task of finding a stable LC, because RRE, schooled in defence requirements for reliable components, appreciated that consumers also would not tolerate short-lived equipment. All available LCs had serious shortcomings. Schiff’s bases gave erratic results, and stilbenes, more recently proposed, were degraded when exposed to ultraviolet radiation.

The solution did not come immediately. Hull worked first on carbonate esters, then on sulphonate and carboxylic esters. They tried purifying samples of Schiff’s bases, to see if the short device life was linked with impurities, and, when this failed, moved to stilbene esters and cyano-Schiff’s bases. All efforts were leading nowhere, and Gray was now becoming frustrated. He decided to take a step back and see if the materials had a common fragile feature. Table 1 shows the position in mid-1972.

**Table 1.** Early liquid crystal families.

family	central link	problem
Schiff’s base	–CN=N–	hydrolytic cleavage
stilbene	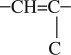	UV instability
azo	–N=N–	oxidation, isomerization
ester	–CO.O–	nucleophilic attack
tolane	–C≡C–	UV instability
azoxy	–N=N(O)–	yellow colouring

Gray realized that one common feature was the central linking group. It would be possible to have a biphenyl structure, but this was unlikely to give a practical working temperature range. Nevertheless, an appropriate end group might give a reasonable range, and they knew from their earlier research that –CN gave a strong positive dielectric anisotropy, crucial for TN devices. The proposed structure was


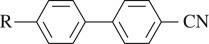


R was chosen to be an alkyl or an alkoxy, essentially the cyano-Schiff’s bases they had worked on earlier, minus the central linkage. After deciding on the way forward, and making some of the necessary precursors, in August 1972 Gray and his colleague John Nash left to attend the International Liquid Crystal Conference at Kent State University, in the USA.

They left with some reluctance, for their recently qualified PhD graduate, Ken Harrison, was ready to attempt the preparation of pentyl-cyano-biphenyl (5CB) and pentyloxy-cyano-biphenyl (5OCB). They returned to a scene of great excitement, for both materials had been made and found to be LCs. 5CB showed a nematic phase from 22 to 35°C, and 5OCB worked from 48 to 69°C. Even more exciting were the results of stability tests at Malvern. The resistivity and the transition temperatures of both materials were unaffected by long exposure to a damp atmosphere, whereas Schiff’s bases became unusable after a few hours. However, this was just the start, because now they must find a mixture that met the temperature requirements, −10 to 60°C. Six alkyls and six alkoxys were then synthesized, and a number of mixtures of these were tried, but the best combination had a range only from −3 to 52°C. They needed to design complicated eutectic systems, but it would have taken far too long to plot eutectic diagrams for all promising combinations.

A crucial contribution was then made by Peter Raynes, who had joined the RRE Displays Group a year earlier, fresh from a PhD on superconductivity. He realized that the Schroeder–Van Laar equation for binary eutectics might be extended to predict mixture properties from thermodynamic data for the individual materials. However, the accuracy was not high enough, and Raynes then developed a more accurate semi-empirical method, which proved ideal. This was so useful commercially that it was not put into print for some years (Raynes 1980). Melting points of eutectic mixtures were then predictable to within 5°C, and clearing points, the change from nematic to isotropic behaviour, to within 2°C (Hulme *et al.* 1974). Raynes predicted that no mixture of biphenyls would operate well below zero. Gray then reasoned that adding a terphenyl component would give a wider range mixture, and though terphenyls were difficult to make, they proved to be the solution.

**Figure 8.**
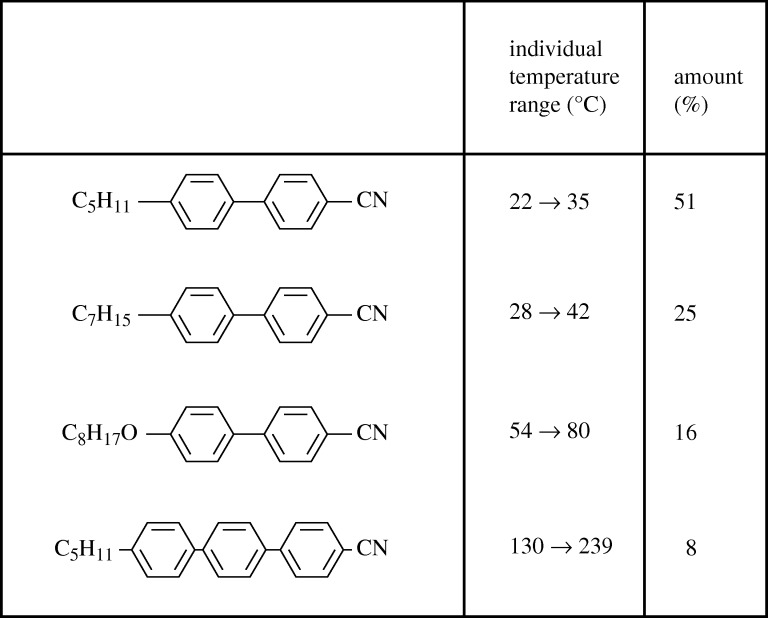
Composition of eutectic LC material E7.

Meanwhile, production processes of pure biphenyls had been perfected at Poole, where Ben Sturgeon, the Technical Director of BDH, had made inspired contributions, and before long BDH was selling biphenyl eutectics widely, for though their temperature range was not ideal, their stability was very attractive. Late in August 1973, Raynes made a four-component eutectic that had a range of −9 to 59°C. It was called E7, and the composition is shown in figure 8. In almost all respects it met the specifications put to RRE by manufacturers of watch displays (table 2).

**Table 2.** E7 as a display material.

property	requirement	E7
nematic range	−10°C to approx. 60°C	−9°C to 59°C
threshold voltage	less than 1.0 V	1.3 V
resistivity	more than 5×10^10^ Ω cm	5×10^10^ Ω cm
response time	less than 50 ms	35 ms
colour	none	none

**Figure 9.**
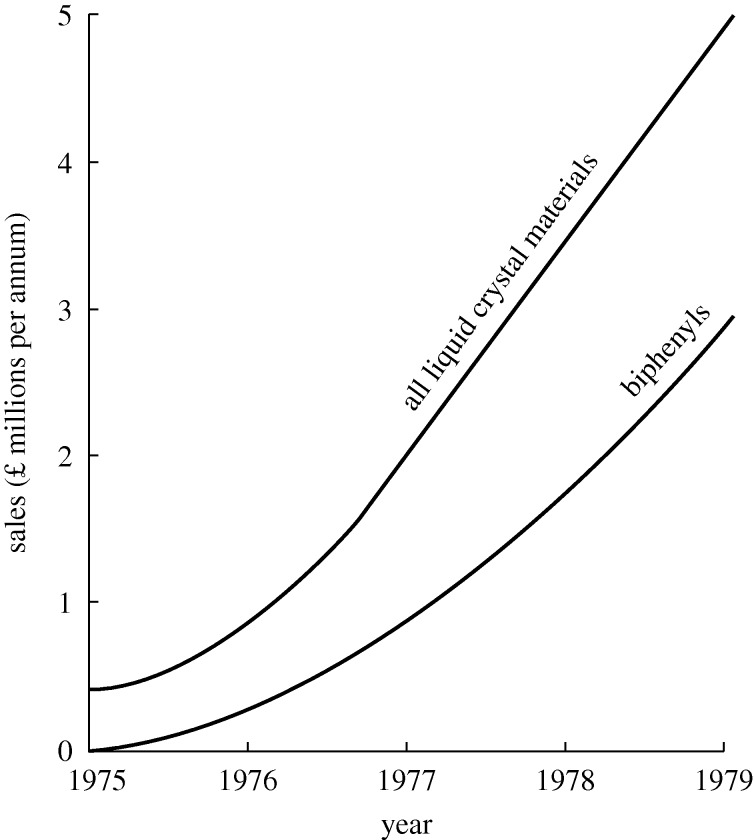
The market for liquid crystals, 1975–1979.

E7 could be said to be the saviour of the LC industry, for it was invented at a time when LCDs were suspected of being inherently unreliable, and it remained the preferred material for many years. The UK Ministry of Defence (MoD) chose a restricted licensing strategy, and originally only BDH and H-LR could sell biphenyls. Rapidly they dominated the market. By 1977 BDH were the largest manufacturers of LCs in the world (figure 9), and biphenyls had become their largest-selling product. Less than five years earlier, the company had never made an LC.^3^

I should not give the impression that the biphenyls had no rivals. The German company Merck Chemicals had made LCs for academic users since 1904, had increased production to meet commercial demand in 1968, and commanded the market before biphenyls appeared. They did not remain idle. In 1973 they bought BDH, and, not wishing to disturb the close relationship between BDH, Hull and the MoD, ran parallel centres in Poole and Darmstadt. Darmstadt was stimulated by the competition, and they conceived their own stable family, similar to alkyl-cyano-biphenyls, but with a cyclohexane ring substituted for one phenyl ring. This family, known as the phenyl-cyclohexanes (PCH), became second in importance to the biphenyls, having a lower viscosity and some very favourable optical properties (Eidenschink *et al*. 1977).

There are many physical parameters of LCs that control the electro-optical behaviour, but the most important for displays are the elastic constants and the rotational viscosity. Table 3 gives the room-temperature values for E7 for the splay (*k*_11_), twist (*k*_22_) and bend (*k*_33_) elastic constants and the viscosity (*η*).

**Table 3.** Parameters for LC material E7.

*k*_11_ (×10^−12^ N)	*k*_22_ (×10^−12^ N)	*k*_33_ (×10^−12^ N)	*η* (cP)
10.70	10	20.70	38

The threshold voltage for switching, *V*_T_, is given by
4.1
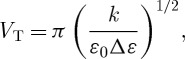

where
4.2
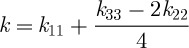

and

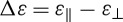

is the difference between the dielectric constants along and across the long molecule. Since Δ*ε* for E7 is 13.8, the threshold voltage will be just under 1 V.

It can be seen that *V*_T_ is independent of the viscosity and the cell thickness (*d*), but the time constants depend on both. We have
4.3
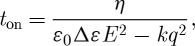

where *E*=*V*/*d* and *q*=*π*/*d*, and
4.4
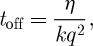

so from equation (4.1)
4.5
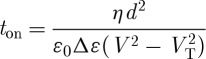

4.6
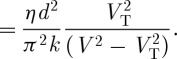

For a 10 μm thick cell, *t*_off_ is about 40 ms, satisfactory for watch and instrument displays, but marginal for video presentation. Since 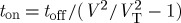, a rapid switch-on is readily available at higher voltages.

The visual appearance of a TN cell depends strongly on the angle of view, and both the viewing angle and the contrast ratio came under criticism as the possibility of major markets became apparent. Major advances were made, both in the cell configuration and in the LC materials. A big step forward was the idea of increasing the twist from 90° to 270°. This supertwist nematic display (STN) was proposed and patented in 1982 by Waters & Raynes (1982) at RRE, and independently patented a year later by the Brown Boveri group, led by Terry Scheffer (Amstutz *et al.* 1983), afterwards ably assisted by Jurgen Nehring. STN gave the steep threshold necessary for passive matrix displays, and the response time and angle of view were similar to the simple TN device (Scheffer 1983; Waters *et al.* 1983). It became the preferred display for instruments and lap-top computers, and lost ground only when the production of TFTs over large areas was perfected. The STN display was patented and widely licensed by the MoD, and yielded royalties of over £100 million, the largest return for any MoD patent.

More radical changes to the TN mode were also introduced. Soref (1972, 1973), at Beckman Instruments and Sperry Rand, had proposed in 1972 displays using circular polarizers with interdigitated electrodes on just one of the glass surfaces. The concept of interdigitated electrodes was improved by the Freiburg Fraunhofer Institute, which invented the in-plane switching (IPS) display in 1990 (Baur *et al.* 1990; Kiefer *et al.* 1992).

The electrodes are on the same cell surface, and, in the absence of a voltage, the LC molecules lie parallel to the surfaces, which have the same rubbing direction, so there is no effect on polarized light. Application of a field between the electrodes induces a rotation on that cell surface, and a twist between the two surfaces. However, fringe fields and the effect of tilt make the operation more complicated, and can lead to increased response time. Moreover, each pixel needs two switching TFTs, and in early versions this reduced the transmittance. IPS was studied by a number of laboratories in the 1990s, notably Hosiden, NEC and, particularly, Hitachi (Ohe & Kondo 1995; Ohta *et al*. 1996). There are now a number of variants in commercial production.

**Figure 10.**
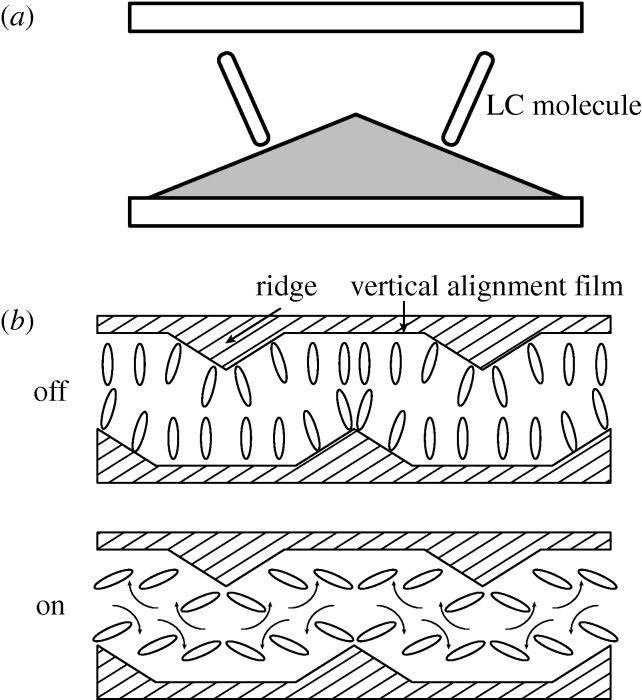
Operating principle of Fujitsu MVA-LCD (after Koike & Okamoto 1999). (*a*) Basic concept. (*b*) Basic cell structure.

Though TN mode devices showed clear advantages over dynamic scattering, several laboratories pursued other LC effects in the 1970s. Fred Kahn at BTL proposed in 1971 a switching effect based on negative nematics aligned homeotropically, i.e. at 90° to the cell walls, so that the electric field was parallel to the long axis of the molecules, the cell being placed between crossed or parallel polarizers. Application of the field will then cause the molecules to rotate through 90°, and the transmission through the cell will change (Kahn 1971, 1972). Kahn showed that *V*_T_ was given by equation (4.1), with *k*=*k*_33_. For the LCs he used, *V*_T_ was 3 V, and the birefringence increased steadily as the voltage was increased to 20 V. Though this seems a simple mode, the alignment requirements are severe. The homogeneous alignment used in TN cells is readily obtained by rubbing the glass surface in one direction. This creates microgrooves, and the long molecules lie in them. For Kahn’s vertical alignment (VA) mode, it is necessary not only to persuade the molecules to lie at 90° to the surface, but also to impose a slight tilt, to give a source of defined anisotropy. This proved difficult to achieve over areas practical for displays, and exploitation of VA awaited the sophisticated control over LC that developed during the next 20 years. A number of improvements were then proposed, one of the most effective being the Fujitsu multi-domain vertical alignment (MVA) mode (figure 10).

In the off state, the molecules adopt a more-or-less homeotropic structure. When voltage is applied, within each domain the molecules align orthogonally to the raised structures, which are actually pyramids, so the light is channelled into a cone, giving a wide angle of view.

**Figure 11.**
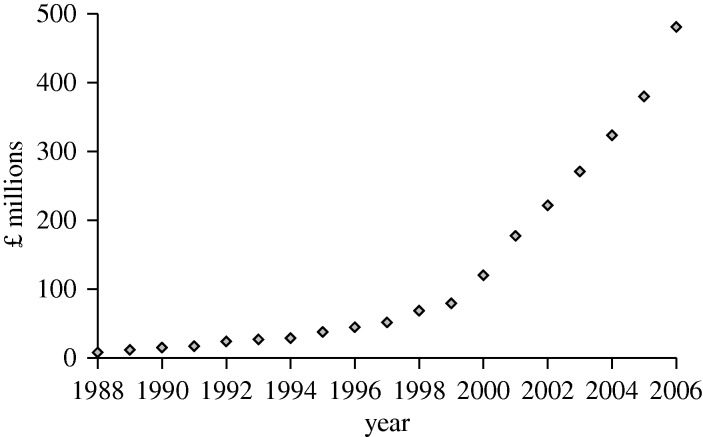
Merck sales of liquid crystals.

The early thirst for LCs shown in figure 9 has not diminished. In 1979 world sales were £5 million. In 2006 Merck, the dominant supplier, had sales of £500 million (figure 11) and their provisional figures for 2007 exceeded £700 million. Two other large suppliers, Chisso and Dai Nippon Ink, are anticipated to have sales of over £300 million, making a sales total of at least £1 billion in 2006. In 1979 sales were measured in grams. Today the measurement unit is tonnes.

Naturally, this growth has had to be served by much R&D on materials to give better display performance. As I noted earlier, the most important parameters to consider in designing an LC for displays are the temperature range of the nematic phase, the magnitude of the elastic constants, the ratio of the bend constant *k*_33_ to the splay constant *k*_11_, and the viscosity *η*. Also relevant are the magnitude and sign of the dielectric anisotropy Δ*ε*, and the magnitude of the birefringence Δ*n*.

**Figure 12.**
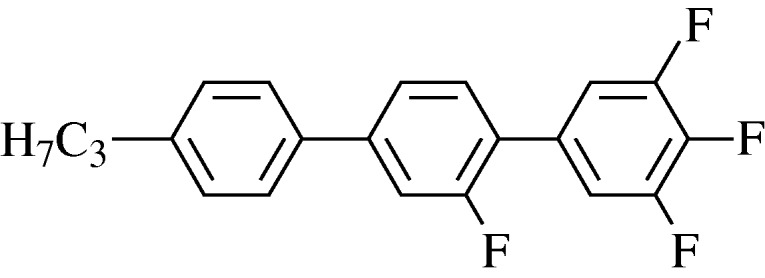
A Merck superfluorinated terphenyl.

Though biphenyls and phenyl-cyclohexanes served the LCD industry well during the first 15 years of development, there were obvious deficiencies in the display appearance and multiplexing capability. One serious problem was the resistivity, insufficiently high for large displays. LCs are insulating, but that is a relative term, and to ensure that the pixel voltage does not drain away in active matrix applications, the resistivity must be very high, above 10^12^ Ω cm, and that rules out some otherwise promising families. Another problem was the slow switching speed, with a failure to follow fast-changing images. The simple remedy of reducing viscosity led to both a smaller operating temperature range and a reduction in the dielectric anisotropy, giving a higher switching voltage. After much research at Hull University and Merck, the inclusion of fluorine groups was shown to give much improved performance (Gray *et al.* 1989; Reiffenrath *et al.*1989*a*,*b*; Coates *et al.* 1993). It should be noted that commercial LCs now are mixtures of from 10 to 30 individual molecules, but a typical core material is shown in figure 12. This material has a high Δ*ε* of over 17, satisfactory for both IPS and TN modes (Kirsch 2004).

**Figure 13.**
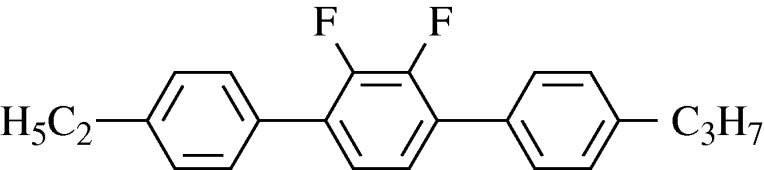
A Merck terphenyl with high negative dielectric anisotropy.

The design of materials for vertically aligned nematic (VAN) mode poses new problems, since they must have a large negative Δ*ε*. It was known that lateral polar substituents would lead to a dipole moment orthogonal to the long axis, and again fluorine was the preferred substituent. The most useful materials are difluorophenyl derivatives and more recently the difluorindanes have been invented, giving a Δ*ε* as large as −8. VAN switching times are longer than for IPS or TN, and the cell spacing has to be as small as 3 *μ*m. This, in turn, calls for a larger value of birefringence, and this often results in high viscosity. A good compromise is found in the terphenyl shown in figure 13, which has Δ*ε*=−2.5, Δ*n*=0.23 and *η*=90 cP (Pauluth & Tarumi 2004). Additional fluorine substituents give larger negative values of Δ*ε*, but the viscosity is increased by a factor of three or more.

#### (ii) Electrophoresis

**Figure 14.**
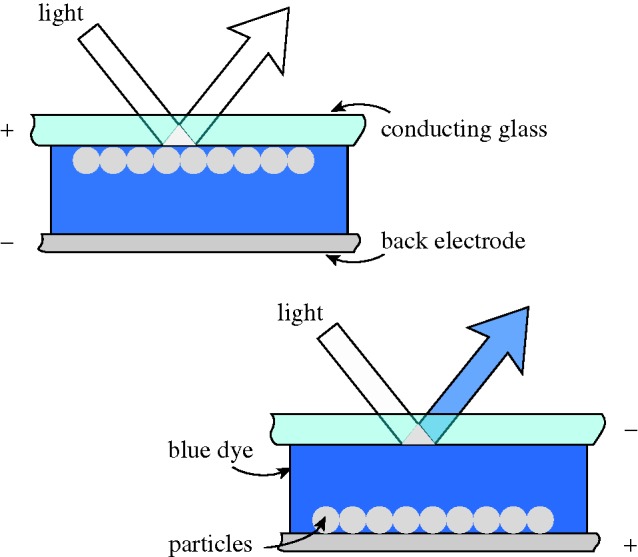
Principle of electrophoresis.

The motion of solid particles in a liquid medium under the action of an electric field has been studied for many years. It is an easy and inexpensive technique, and is often used for species separation and for particle deposition on substrates, as in painting large areas uniformly. The system becomes a display when the particles have a colour that contrasts with that of the liquid. Typically, white TiO_2_ particles in blue ink give a most impressive high-contrast image (figure 14).

Electrophoresis (EP) was one of the early candidates for flat panels, but research revealed several major disadvantages. The most serious were gravitational settling, flocculation of the individual particles into clumps and electrode staining. Staining appeared in time because a field sufficiently high to move the particles towards an electrode would not always be sufficient to extract it. Clumping gave a variation in mobility, so that a clump would have a slower response time than a single particle. In any case, the drive voltage was higher than for LCDs, and the response time slower. Some of the problems were summarized by Hopper & Novotny (1979) from Xerox.

The effect was thought unpromising by the display community for several years until the Nippon Mektron company suggested that microencapsulating the particles would solve many of these problems (Osamu *et al.* 1987). There followed refinements of this technique (Jacobson *et al.* 1997; Comiskey *et al.* 1998) until today it is regarded as a leading candidate for electronic paper, which will be considered later. The more general application to displays is less obvious, because transmissive effects are poor.

#### (iii) Electrochromism

Striking colour changes are seen in some solids and liquids on the application of an electric field. The first attempt to exploit such effects came in 1948 from Sziklai (1948), a prolific inventor at RCA, who patented an electrically controlled colour filter for TV cameras. The realization that this gave the foundation for flat-panel displays came almost 20 years later, but then a number of laboratories produced simple prototypes. Early in the field of electrochromic solids was American Cyanamid (Deb & Shaw 1966; Deb & Witzke 1975) and ICI, which studied effects in organic liquids in-house (Kenworthy 1971) and sponsored research on oxides at Imperial College (Green & Richman 1974; Green 1976). The Philips Research Laboratory, Eindhoven, made practical displays based on viologen derivatives (Van Dam *et al.* 1972; Schoot *et al.* 1973).

The basis of the effect is a reduction–oxidation reaction, and for many years the favoured materials were either simple salts or polymeric forms of bipyridyls or, for the ‘solid’ displays, transition metal oxides. The oxide displays used a fast-ion conductor as the electrolyte. The most-studied liquid system used viologen derivatives, and operated by combining a plating reaction with a dye reaction, so depositing a violet film on the transparent electrode. One advantage of electrochromism is that it has memory, so that power is required only when the data change. Unfortunately, this advantage is temporary, since the effect is not absolutely reversible, and in time the electrodes become stained. Early viologen displays had a short lifetime because of recrystallization. The first solid displays used WO_3_, but the response time was over a second. Combinations with Mo, V and Nb gave different colours, and a move to liquid electrolytes improved the response time to some tens of milliseconds, but the lifetime was too short for general adoption. There might have been more sustained interest if the effect had a threshold, but, as it was, multiplexing was difficult, and there was therefore little advantage over the alternative effects.

New life has been given recently to the science of electrochromism by the appearance of two important applications, electronic paper and variable transmission windows. This interest has stimulated materials research, and electrochromism in polymers has made much progress. However, the short life, about 10^6^ cycles, and the long response time of several hundred milliseconds, remain as problems to be overcome before electrochromism can play a part in dynamic displays.

#### (iv) Electrowetting

**Figure 15.**
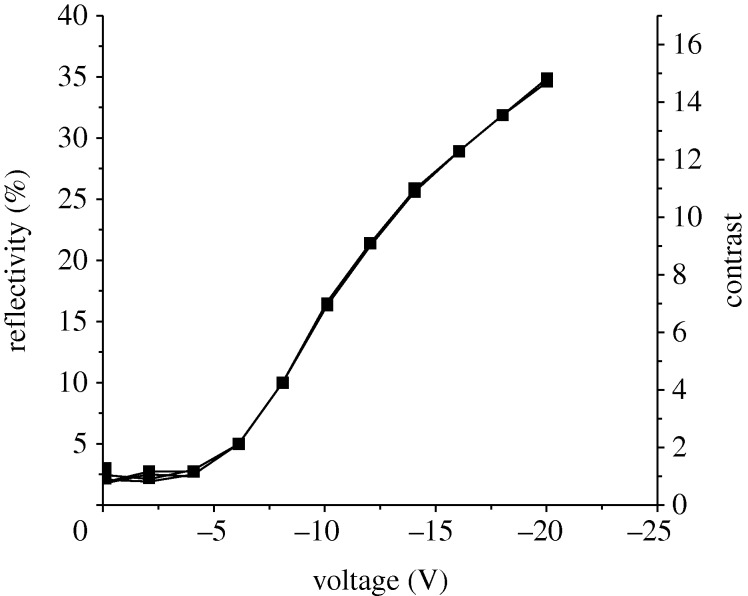
Electrowetting characteristic (after Hayes & Feenstra 2003).

For a brief period after 1980 it appeared that the tide on the wisdom of flat-panel development was turning in BTL as the influence of the opposing senior managers diminished, and a novel approach was published. This was based on inducing motion in a fluid by the application of an electric field. Gerardo Beni and Susan Hackwood showed that a porous solid could be filled with a liquid of the same refractive index, so that it was transparent. If the liquid was then extracted from the solid, the pixel would be diffusely reflecting. They found that visible changes occurred at applied voltages of about 1 V with response times of a millisecond (Beni & Hackwood 1981). This promising start was not pursued for long at BTL, but the technology was exploited in microfluidics and tunable liquid microlenses. The circle was completed over 20 years later when Robert Hayes and Johan Feenstra, of Philips Research in Eindhoven, proposed an electrowetting display of a rather different type. The pixel was a white surface, which in quiescence was covered by a thin film of coloured oil. Application of 20 V caused the film to curl into the pixel corner, causing a marked change in reflectivity (figure 15). For small pixels the response time was about 10 ms (Hayes & Feenstra 2003). The reflectivity is much greater than that of LCDs, and approaches that of paper. In transmission the technique may offer less advantage, but the application to electronic paper is obvious.

Understanding of electrowetting is incomplete, but some extensive reviews are now available (e.g. Mugele & Baret 2005). Only certain surfaces demonstrate the theoretically predicted behaviour, but as the technique acquires commercial importance, it is likely that more detailed physical models will emerge.

### (b) Emissive displays

**Figure 16.**
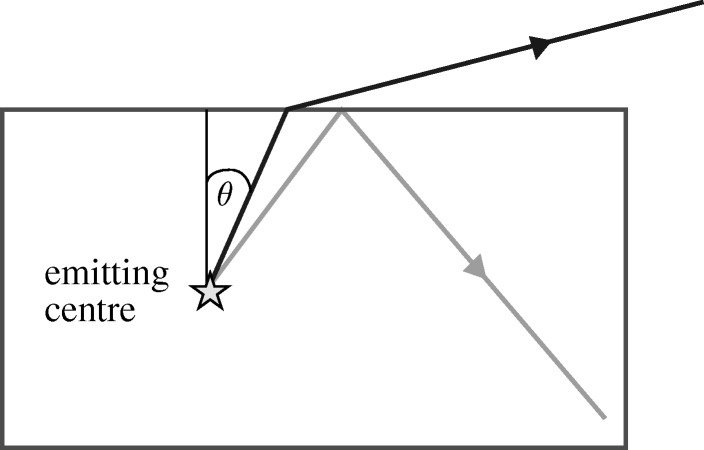
Frustrated total reflection.

The exploitation of emissive effects in solids raises an immediate problem. If the solid surface is planar and the emitted light is incident on the interface at an angle greater than sin^−1^(1/*n*), *n* being the refractive index of the solid, the light cannot emerge (figure 16).

The loss is serious for many semiconductors used in LEDs. For example, *n* is 3.66 for GaAs, so the critical angle *θ*_c_ is only 16°, and only 4 per cent of the energy radiated into a hemisphere reaches the outside world. Fortunately refractive indices decrease as energy gaps increase, but even so only 10 per cent of the radiation from a semiconductor emitting at visible wavelengths would emerge. It is possible to reduce the loss in an LED by shaping the surface as a dome or Weierstrasse sphere (Carr 1966), but that would not be simple for a display, with perhaps 10^6^ emitting pixels each less than 1 mm^2^.

#### (i) Inorganic solid emitters

**Figure 17.**
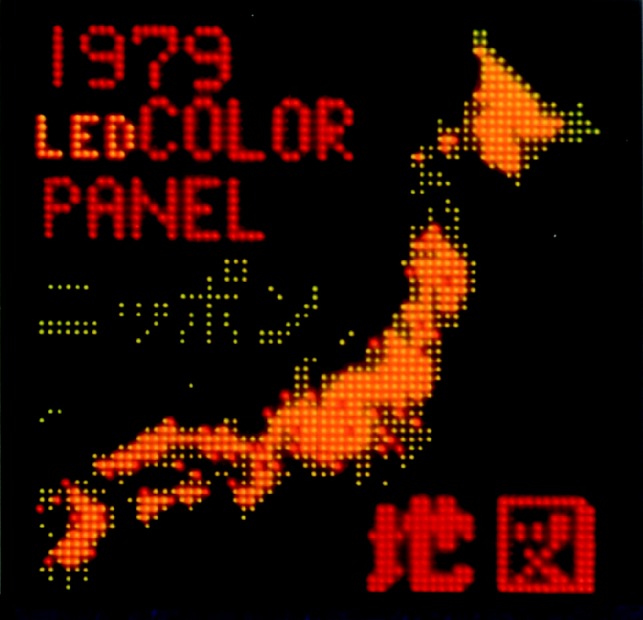
An early *tour-de-force* in LED assembly: multi-colour LED flat-panel display with 64×64 picture elements.

The conversion in a solid of electrical energy into light was first observed in 1907 by Henry Round, of Marconi, who saw yellow light emitted from a SiC crystal when he applied a voltage to it (Round 1907). Round published his discovery in an admittedly obscure journal, and it attracted little interest. In 1923 Oleg Lossev at the Radio Laboratory in Nijni-Novgorod, unaware of Round’s work, repeated the experiment, and published in higher-impact journals (Lossev 1923, 1928). Diode electroluminescence (EL) is therefore known as the Lossev effect. LEDs are now universal, and it was natural in the early days of flat-panel R&D to consider their relevance. LEDs are made with high density on single-crystal substrates, and the size of the substrates is rarely more than a few centimetres square. Assembling substrates on a large area and handling the interconnections would be expensive, and there has been no serious attempt to fabricate a TV display for sale (figure 17). Moreover, at a time when subtractive effects were making slow progress, no efficient blue LED was available. Today a full range of colours is available, and huge LED displays are seen in many sports stadia.

**Figure 18.**
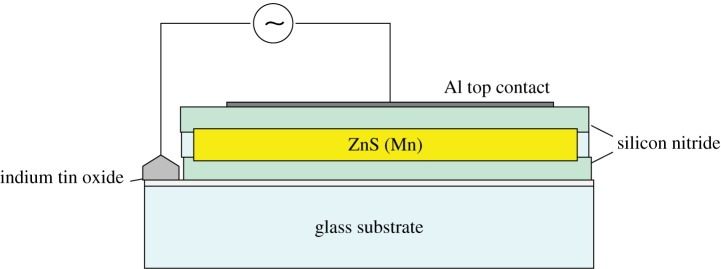
Thin-film AC electroluminescent panel construction.

A more practicable approach to EL had actually come in 1936, when Destriau (1936) applied an AC field to a capacitor made with ZnS powder immersed in a dielectric. His device and the subsequent industrial versions were not used extensively because of their short life at high brightness. Lower-brightness panels, made by firing a coating containing ZnS particles on to a steel plate, were marketed by GTE Sylvania, but their application was as night-lights, not displays. The modern version of the Destriau panel, shown in figure 18, is made entirely from thin films, avoiding most of the degradation processes of powders.

Above a critical field, typically 10^6^ V cm^−1^, insulating layers of ZnS will show a rapidly increasing current with voltage increase, and they simultaneously emit light. Generally manganese centres are incorporated in the ZnS to obtain yellow emission, but other centres, such as rare earths, can also be used. The problem is to operate the layer close to breakdown without risking current runaway. In the AC device shown, only a limited amount of charge is permitted to pass through the ZnS, and this amount depends on the capacitance of the dielectric. Clearly the dielectric must not break down, nor must it demand too high a fraction of the voltage drive. To ensure that the field in the ZnS is above the threshold required for light emission *E*_T_ and the dielectric is kept below its own breakdown field *E*_DB_, the inequality
4.7
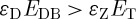

must be maintained, where the subscripts refer to dielectric (D) and ZnS layer (Z) respectively. We have, for applied voltage *V*_A_,
4.8
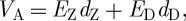

with *E* representing field and *d* thickness. The *E*_Z_*d*_Z_ product must dominate the r.h.s. of this equation to give high efficiency at as a low a voltage as possible. Charge continuity demands constancy of *Eε* products, giving us three conditions for the dielectric films.

— The dielectric must be thin.
— The dielectric constant must be high.
— The breakdown field must be high.

Typically, for a dielectric with *ε*=10, *d*=2000 Å and breakdown field of 3×10^7^ V cm^−1^, operation at 250 V AC is satisfactory. These values must be achieved simultaneously, i.e. the high breakdown field is needed in layers as thin as several thousand ångströms, otherwise the efficiency will be low. I must emphasize the need to optimize efficiency, which is, at best, only about 0.5 per cent. The external efficiency is reduced by light trapping within the thin transparent film, which can easily give a loss of an order of magnitude in such high refractive index systems.

Such panels were made in 1974 by Sharp, and a few years later by Tektronix. They each faced the breakdown problems with some success, but the only reliable efficient phosphor available was ZnS doped with Mn, giving an excellent yellow, but no good blue or red. A spin-off company from Tektronix, Planar Systems, did report blue-green emission from SrS:Ce, but, in practice, only small AC electroluminescent (ACEL) panels were ever produced in number because of the competition from LCDs.

**Figure 19.**
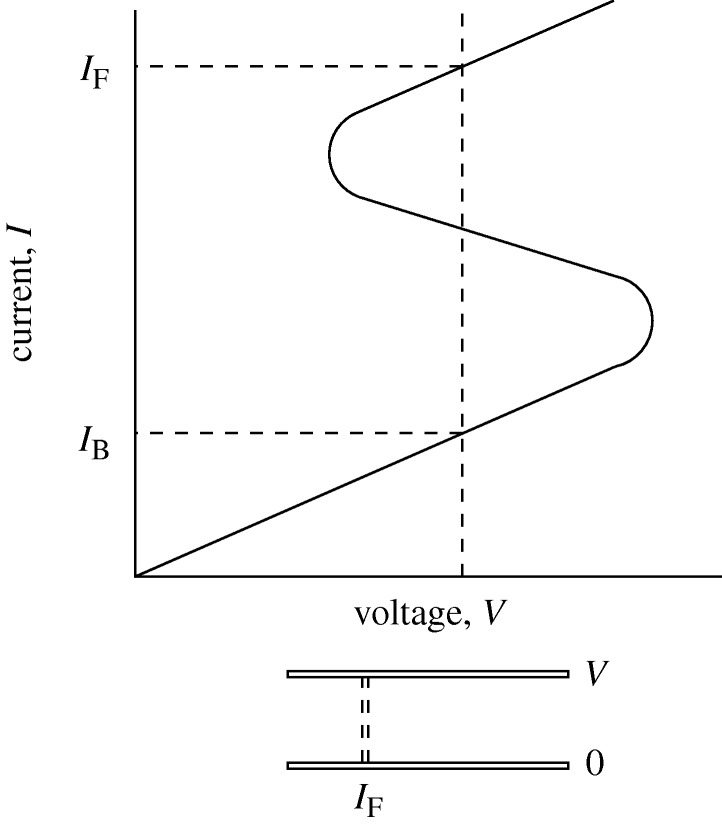
Filamentary breakdown due to S-type negative resistance.

A major defect of the ACEL panel, which has excellent appearance, and can be multiplexed to a high level, is the high voltage of operation. Special circuits must be manufactured, and these add much to the cost of the panel. It should be simpler, and cheaper, to drive the panels DC, but there are severe problems in driving a large-area device near to breakdown without risking catastrophic failure. A film that is non-uniform in thickness, composition or geometry will obviously break down at weak points, either where the layer is abnormally thin, or where there is field amplification at edges or points. If we construct a uniform film, there is still the possibility of an S-shaped negative resistance (figure 19), which leads to current concentration in filaments, with consequent breakdown due to local heating.

It follows that stable DC thin-film EL over a large area requires a layer that adds a current-limiting resistance in series, the DC equivalent of the AC dielectric. This must be done without adding appreciably to the operating voltage, with a consequent reduction in the already low efficiency. The problem is not simple.

Successful DC electroluminescent (DCEL) panels were made, but these relied on phosphor powders packed closely enough together to give conduction. In addition, the ZnS:Mn powder had Cu added, and this was often in the form of a surface-rich layer. In general, however, panels made in this way had low brightness, low efficiency and short life. A major step forward was made by Aron Vecht, who had led research on EL at Associated Electrical Industries, Rugby, but left in 1968 when the company merged with GEC, and the responsibility for EL passed to the Hirst Research Centre, Wembley. Vecht and some key members of his team moved to Thames Polytechnic, later to become the University of Greenwich, and there he invented an ingenious way of creating stable DCEL panels (Vecht 1970; Vecht & Werring 1970).

The ZnS particles were coated with Cu, immersed in a polymethylmethacrylate or nitrocellulose binder, spread over a glass plate previously coated with a transparent conducting film, and having an Al or Cu electrode applied to the surface to act as a cathode. The powder concentration is so high that a conducting path exists between the top cathode and the transparent conductor.

**Figure 20.**
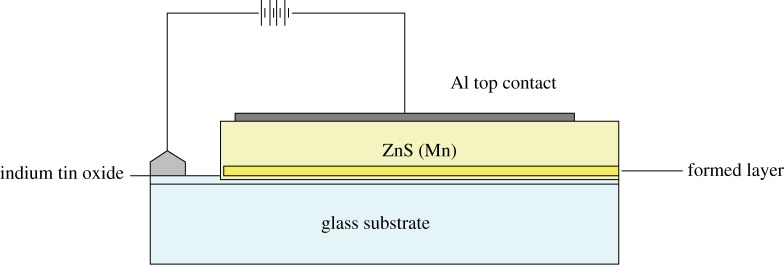
A formed DC electroluminescent panel.

The emitting region is formed by passing a current high enough to heat the panel, and over a few hours the panel impedance increases. The applied voltage is steadily increased from a few volts to a maximum value, typically 80–100 V, to maintain the consumed power approximately constant. The electric current passing through the panel produces a narrow high-resistivity light-emitting barrier (typically a micrometre thick) near the positive front electrode film, and it is the gradual formation of this region that causes the increase in panel impedance. Presumably this region is formed because Cu diffuses away from the anode (figure 20).

By 1980 Vecht had improved the performance of DCEL panels enough for them to be considered for consumer applications. When driven at 100 V, they gave an initial brightness of 300 cd m^−2^, and had a life to half-brightness of over 15 000 h (Vecht 1982). Two problems prevented wider adoption. Though the Mn emission spectrum was broad enough for good reds and greens to be obtained by filtering, no efficient blue emitter was discovered. Rare earths did give a good narrow blue line, but the solubility was too low for the emission to be useful. The other problem was the cost of 100 V driver chips, and this made both ACEL and DCEL uncompetitive. However, that situation changed when the potentialities of organic materials for EL became apparent.

#### (ii) Organic solid emitters

There was little interest in EL in organic materials for some years after Destriau’s work, but in 1953 one French group, led by Bernanose (1955), and in close collaboration with Destriau, observed ACEL in acridine and carbazole at voltages up to 2 kV and frequencies of 50 Hz. In 1960 Edward Gurnee and Reet Fernandez of Dow Chemicals showed that a variety of organic compounds could be used, and found that most light was emitted when the phosphor was a host containing a doping agent or activator, and the colour depended on the combination. A typical phosphor had anthracene as the host, with 0.1 per cent tetracene as the activator. The EL cell operated off 800 V at a frequency of 2 kHz (Gurnee & Fernandez 1960). By 1962 Gurnee had added a thin insulating layer in the cell construction, thereby reducing the operating voltage to 110 V and the frequency to 60 Hz (Gurnee 1962).

At about the same time we saw the first DCEL organic devices, when Martin Pope^4^ used a thin crystal of anthracene with NaCl solutions as electrodes and applied voltages of 400 V or more. It was apparent that the only way of passing current through a material that was insulating at low voltages would be to design injecting contacts, and the experiments had limited success (Pope *et al.* 1963). Dresner & Goodman (1970*a*,*b*) made some progress in 1970 with a tunnel cathode, but the operating voltage was still nearly 100 V. The next forward step remained unacknowledged for several years, being described in a patent by Roger Partridge, of NPL, in 1975 (Partridge 1975). He set out clearly the device design for an organic LED (OLED): a low-work-function cathode, to inject electrons, a high-work-function anode, to inject holes, and, between them, a luminescent organic film, which he specified as being a polymer, such as polyvinylcarbazole. Partridge’s patent is specific on the materials and thicknesses of the various films, but he did not submit his work for publication until 1981, and his results were not generally available until mid-1983 (Partridge 1983*a*–*d*). The delay was not, of course, Partridge’s responsibility. The Government department to which NPL reported, the Department for Industry, wanted to preserve publicity until they could identify exploitation routes. The secrecy was compounded by entitling the patent ‘radiation sources’, a catch-all phrase (R. H. Partridge 2009, personal communication). This strategy would have been admirable if the Department had then used its resources to secure industrial collaborators, but such efforts as it made were unsuccessful. As a result, Partridge’s claim for priority has only recently been acknowledged generally. He was the first to design and make polymer LEDs (PLEDs) with injecting electrodes, though he did not specify the advantages of using conjugated polymers, one key to later development.

Before Partridge’s paper appeared, others had made important contributions. Significant was the work of Gareth Roberts’ group at Durham University, which first obtained EL in 1979 from a Langmuir–Blodgett film of anthracene (Roberts *et al.* 1979), and in 1982 used a vacuum-deposited film of anthracene less than 1 μm thick, and saw blue light at a voltage of 12 V (Vincett *et al.* 1982).

**Figure 21.**
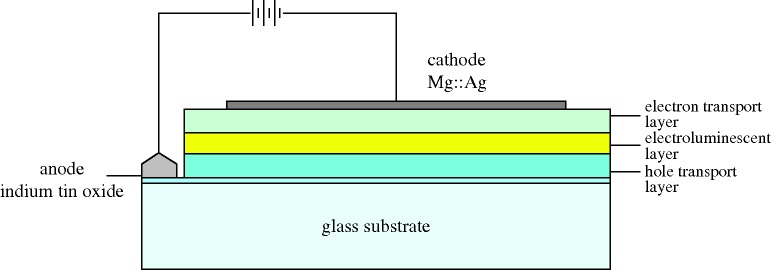
Organic LED schematic.

The potential of the field was now apparent, and many groups were working on EL in small molecules or polymers. Noteworthy was the group at Kyushu University, which seem to have been the first to obtain results from layered devices, using cyanine dyes (Era *et al*. 1985), and then perylene films (Hayashi *et al.* 1986). Members of this group, Chihaya Adachi, Shizuo Tokito, Tetsuo Tsutsui and Shogo Saito, specifically prescribed the structure necessary for low-voltage efficient OLEDs, shown as a schematic in figure 21. Early in 1988 they reported three-layer devices emitting blue, green and orange-red light from anthracene, coronene and perylene respectively (Adachi *et al.* 1988).

The device is made on a glass substrate coated with ITO. ITO has a high work function, and acts as the anode, injecting holes. The next layer must transport holes (hole transport layer, HTL), but should also inhibit the passage of electrons. Next to this layer is the electroluminor, where the holes and electrons will recombine, and there is then an electron transport layer (ETL), inhibiting hole passage. The final metal cathode has to be a low-work-function metal, such as Mg:Ag. This structure is now fairly standard, though in some designs one material can perform two functions. The physics underlying the work of the Kyushu group cannot be faulted, but their embodiment did not perform well, with a very low efficiency, a drive voltage of 50 V, and a life of only 5 h.

Adachi had been a PhD student at Kyushu, and he has continued to make significant contributions to the field at various laboratories since then, returning to Kyushu 4 years ago. His early collaborator at Kyushu, Tsutsui, remained there throughout his career, again making significant contributions until his recent retirement. Their work has, perhaps, received less acknowledgement than have the groups linked with industry, but both have certainly been major forces in OLED development since its inception.

Ching Tang had worked at Eastman Kodak in the early 1980s on organic EL cells similar to those developed for ACEL in ZnS, described in the previous section. He had substituted tetraphenylethylene or similar organics for the ZnS, held them in a polymer binder, and found that conduction was possible when a layer of a porphyrinic compound was inserted between the anode and the organic emitter. The cell operated at quite low voltages, around 30 V, but the efficiency was well below 1 per cent (Tang 1980). He then abandoned the binder, and in 1987, with Steven VanSlyke, published the classic paper on OLEDs, with electrons emitted from an Mg:Ag cathode into a layer of Alq_3_, tris(8-hydroxyquinolinato)aluminium, with a layer of diamine acting as the HTL. The Alq_3_ layer plays two roles, acting as both the electroluminor and the ETL. Green light was emitted, with an efficiency approaching 0.5 per cent at 5.5 V. The life of the device was 100 h, though the voltage had to be increased steadily to maintain the light output (Tang & VanSlyke 1987). This paper has now been cited over 5000 times, and was followed by a string of publications and patents over the next 20 years. Much of the progress in the field has been due to the work of them and their colleagues at Eastman Kodak.

**Figure 22.**
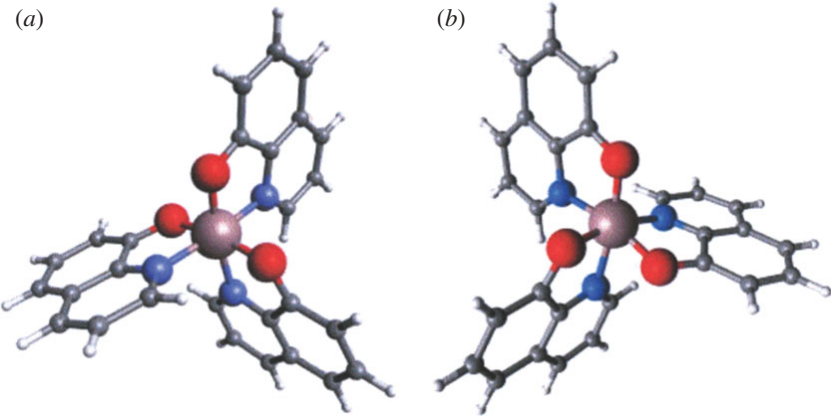
Isomers of Alq_3_: (*a*) meridional; (*b*) facial (violet, Al; red, O; blue, N; after Cölle & Brütting 2004).

Alq_3_ has proved a critical component in most advanced OLEDs since its favourable properties were shown by Tang and VanSlyke. It is a complex molecule that can adopt a number of crystalline phases, but, since it is usually deposited by evaporation, resulting in an amorphous film, control of the composition is not easy. Moreover, though for many years it was believed that there was a single isomer, it is now clear that there are two, one meridional and one facial, shown in figure 22 (Cölle & Brütting 2004). The characteristic green emission is due to the meridional isomer, and the facial, often present as a few per cent in amorphous films, emits in the blue.

PLEDs had remained unnoticed after Partridge left the field. However, a group at Cambridge University, led by Richard Friend, had become interested in the possibility of OLEDs, but believed that the Kodak approach had an inherent problem of structural instability due to recrystallization. They thought that a better luminor would be a polymer, and, in particular, a main-chain conjugated polymer. They noted the ease with which thin films of polymers could be prepared by spin-coating, and in 1990 made simple diodes, with poly(*p*-phenylene vinylene) (PPV), a polymer known to show bright green photoluminescence, spun onto ITO-coated glass, with a cathode of Mg–Ag. The devices emitted green light at 15 V, albeit at low efficiency, and with a life of a few days (Burroughes *et al.* 1990; Friend *et al.* 1990). Their publication attracted much attention, and the approach was followed by a number of laboratories. Alan Heeger, later to receive a Nobel Prize for the discovery of conductive polymers, modified both the PPV polymer and the cathode, and obtained an efficiency approaching 1 per cent (Braun & Heeger 1991).

There was early realization that the simple sandwich was unlikely to provide the environment needed for high conversion efficiency, with equal numbers of electrons and holes available for recombination in the polymer luminor. There seemed to be some resistance to designing a cell with the full gamut of layers shown in figure 21, though Friend, with colleagues including Adam Brown, Jeremy Burroughes and Donal Bradley, did show that an ETL of biphenylylbutylphenyl oxadiazole (butyl PBD) gave improved efficiency (Brown *et al.* 1992). Adachi had previously shown that butyl PBD performed well as an ETL in blue-emitting OLEDs (Adachi *et al.* 1990). It was also appreciated that acceptable device lifetime could be achieved only if the cells were encapsulated to protect them from the effects of oxygen and moisture. This was particularly necessary if Ca was used as a cathode, capitalizing on its low work function. There followed a period of steady progress in both efficiency and lifetime, and the use of a variety of luminors to give a full range of colours.

By the turn of the century both organic (OLED) and polymer (PLED) devices had made such progress that commercial exploitation appeared likely. Efficiencies of both types and of all colours were well above 1 per cent, and some designs were giving life at constant current of over 10 000 h. There was some concern that the drive voltage almost doubled during that period, and this would certainly complicate incorporation in multi-pixel panels. Blue devices were still lagging in performance and lifetime, but it was thought that this problem would yield to increased research. Commercial interest of Kodak had always been high, and in the UK Cambridge University spun out Cambridge Display Technology (CDT) to exploit PLEDs. Many of the world’s electronic companies were beginning to become involved.

Efficiency lagged behind desired specifications for brightness. The refractive loss analysed at the beginning of §4*b* was not easy to overcome, but several remedies were tried. Almost half the loss came because most of the light emitted away from the anode was lost. Composite metal–dielectric layers could reduce this loss. The plane glass surfaces could be etched to give scattering, or low-index aerogels interposed (Smith & Hilsum 2003). Microlenses could be attached to the surface, though this would prove problematic for small pixels on large areas. One remedy that attracted considerable effort was the concept of microcavities. This entails forming Fabry–Perot etalons at each pixel by interposing a dielectric mirror stack between the glass and the ITO. This should give directionality, and in principle enable most of the radiation that is produced by recombination to emerge. There are two obvious problems. One is the need for different mirror stacks for each pixel colour. The second is the need for an appreciable angle of view (Peng *et al*. 2003; Shiga *et al.* 2003).

Though the refractive loss is serious, it does not account for the relatively low internal efficiency. The excitons formed in the process of recombination take either a singlet or a triplet spin configuration, and simple theory indicates that the ratio of these states is 1 : 3. Unfortunately the more numerous triplet states are non-radiative, so that the maximum internal EL efficiency would be 25 per cent. Since an isotropic emitter with the refractive index of PPV or Alq_3_ could emit into the outside world only 25 per cent of the photons produced, the maximum external efficiency could not exceed 6 per cent. Measurements confirmed that the singlet fractions in Alq_3_ were near 20 per cent (Segal *et al.* 2003). The situation was not as serious for polymers, for early measurements showed that the singlet/triplet ratio could be as high as 50 per cent, and detailed theory indicated that the actual ratio would depend on the polymer, and in particular on the energy gap (Wilson *et al.* 2001). There was, nevertheless, good reason to explore methods for reducing or eliminating non-radiative recombination for both OLEDs and PLEDs. Two approaches proved effective.

The first proposal for reducing the effect of the non-radiative triplet states came from Herbert Börner and his colleagues at the Philips Laboratory in Aachen. They filed a patent in 1994 suggesting that the OLED should incorporate a phosphor having emitting triplet states at energy levels available for transfer of energy from the luminor non-radiative triplet states (Börner *et al*. 1995). Though Börner clearly appreciated that this should give an improvement in performance, he gave no details of any gain he actually achieved, and there was no subsequent publication. The concept of exploiting phosphorescence was more clearly stated by the group at Princeton University led by Steve Forrest and including Mark Thompson and Paul Burrows. The group had previously shown that organic devices suffered severe degradation if exposed to the atmosphere during life, and demonstrated the improvements on encapsulation (Burrows *et al.* 1994). They now played the major role in establishing the science of phosphorescent doped OLEDs, or PHOLEDs, and filed a number of patents and published many papers from 1997 onwards (Thompson *et al.* 1997; Baldo *et al.* 1998; Baldo *et al*. 2000). Thompson had moved to the University of Southern California (USC) in 1995, but he still worked closely with Forrest’s group in the search for efficient OLEDs, and their results were exploited via a collaboration with United Displays Corporation (UDC), a company founded in Ewing, near Princeton, in 1994. UDC has grown to be one of the major suppliers of materials to the display industry. The invention of phosphorescent doping played a major role in achieving commercially viable OLED efficiencies, and world efforts are well summarized in Holder *et al.* (2005).

**Figure 23.**
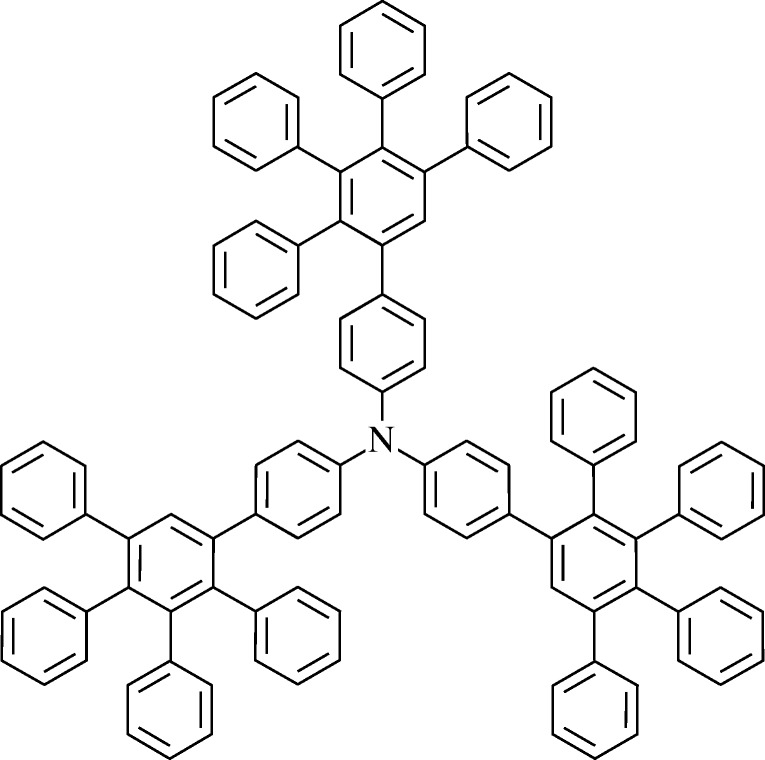
Schematic of a starburst dendrimer.

The second initiative was relevant to PLEDs. In the 1980s, Dow Chemical developed and trademarked Starburst dendrimers, a novel type of three-dimensional polymer with hollow interiors and surfaces that can be closely packed with a variety of molecules (figure 23).

The materials are extremely versatile, spanning many structural and functional applications, and if the right molecules are incorporated, they are efficient phosphors. The team that decided that dendrimers could make efficient PHOLEDs was led by Paul Burn and Ifor Samuel. Burn had earlier been a member of the Cambridge University PLED team, but moved in 1992 to the University of Oxford, hoping to develop conducting dendrimers. Samuel had set up his own group at the University of Durham in 1995, with an interest in light-emitting dendrimers. Together they showed that dendrimers could be designed to give efficient blue, red and green PLEDs, and founded Opsys in 1997 to exploit their ideas (Samuel *et al*. 1998; Ma *et al.* 2002; Burn *et al.* 2007). CDT bought Opsys in 2002, appreciating that dendrimers could be effective in improving the performance of their PLEDs, and could be deposited by spin-coating or ink-jet printing, Some of their current commercial PLEDs incorporate dendrimers.

Another pioneer in this application of dendrimers was Jean Fréchet, who had earlier developed photoresists for integrated circuits, and later used dendrimers for drug delivery and tissue scaffolds (Gilat *et al.* 1999; Adronov & Fréchet 2000).

A development that may well affect the future design of OLEDs came from the Technical University of Dresden, where Karl Leo, Jan Blochwitz and Martin Pfeiffer had been considering the analogy of OLEDs with inorganic LEDs. They thought that the operating voltage of OLEDs was unnecessarily high because of the two blocking layers inherent in all previous designs. Their aim was to make an organic p-i-n diode, with holes injected from the anode into an intrinsic region where they would combine with electrons injected from the cathode. Obviously this entailed controlled p- and n-doping of the appropriate layers. They accomplished this not by adding donor and acceptor elements, but by molecular doping, and in a series of publications from 1998 to 2003 reported that controlled doping was possible (Pfeiffer *et al.* 1998; Werner *et al.* 2003). A spin-out company, Novaled, was founded in 2003, and this has now become a major player in the field, specializing in PIN-OLEDs. Since the improvement comes from the contact layers, advances in either dendrimers or phosphorescents can be exploited in the intrinsic region of the diode, so Novaled works on both polymer and small-molecule LEDs.

The current state of the art is shown in table 4. Some caution is appropriate here. The result from Wei-Sheng Huang, Jiann Lin and Hong-Cheu Lin, in Taiwan, though a striking achievement, may not indicate commercial potential, because life data are not provided (Huang *et al.* 2008). On the other hand, information from companies may not represent the true state of their art, because commercial confidentiality may restrain them from revealing the full extent of their capabilities. The commercial competition is very fierce, and as a result it is difficult to give the composition of the materials favoured for devices, for either POLEDs or OLEDs. In any case, today’s materials are likely to be supplanted in the near future by new formulations.

**Table 4.** Performance data for commercial OLEDs and PLEDs.

	efficiency	efficiency	efficiency	life (h)	CIE
	(Cd A^−1^)	(%)	(l W^−1^)	at 1000 cd m^−2^	coordinates
*red*					
CDT	30			more than 260 000	0.63, 0.37
UDC	28	20		330 000	0.64, 0.36
Kodak	12	8.5		more than 70 000	more than 0.66
Novaled	12	12	10.6	more than 500 000	0.68, 0.31
*blue*					
CDT	9.4			10 000	0.14, 0.14
UDC	9	4			0.14, 0.13
Kodak	8	6.7		10 000	0.14, 0.15
Novaled	9	5.5	8.4		0.14, 0.23
*green*					
CDT	21			110 000	0.29, 0.64
UDC	67	19		250 000	0.38, 0.59
Kodak	30	8.7		65 000	0.25, 0.66
Novaled	95	23	116		0.31, 0.66
Taiwan group	61	17.6	32		0.29, 0.62

There remain several serious hurdles to overcome in the application of these devices. Spin-coating was thought to give PLED manufacture an advantage over OLED sublimation, but colour patterning is not simple, and several companies have concentrated on ink-jet printing. Similarly, mass production of large areas by sublimation is not cheap, and UDC has pioneered organic vapour-phase deposition (OVPD), as used for many years for compound semiconductors (Forrest 2004). They showed that this gave good results for PHOLEDs, and worked with Aixtron, which now sell OVPD commercial production equipment. Kodak, for their part, have developed vapour injection source technology, which they claim is suitable for large-area production, and is more economic than sublimation (Kyoshi 2006).

One important recent development that could either improve the future prospects of flat-panel organic displays or act as a considerable diversion of resources is the realization that organic diodes could be efficient domestic and commercial sources of illumination. Tungsten filament lamps have a light production efficiency of less than 20 l W^−1^, or 3 per cent, and fluorescent tubes give at best 100 l W^−1^ (15%). Since lighting constitutes a high proportion of a country’s energy needs, government interest in this field has grown rapidly, and has been accompanied by generous financial support. It is no surprise, then, to see the major display players establish lighting divisions. The two applications have much in common, but there are significant differences. The most obvious is colour, where the illumination need is for a white, or ‘warm white’ panel, and here progress has been rapid. Many laboratories have reported white LEDs with external quantum efficiencies of 15 per cent, and the University of Dresden obtained an efficiency of 90 l W^−1^, with the prospect of 124 l W^−1^ if measures were taken to reduce the total internal reflection (Reineke *et al.* 2009).

#### (iii) Gas plasma discharge

Light emission from gas discharges is over 200 years old, and the use of inert gases to give specific colours dates back over 100 years. The first attempt to exploit the effect in a display was made 40 years ago, when Don Bitzer, Gene Slottow and their colleagues at the University of Illinois proposed a display panel in which the discharge was restricted to local areas by a glass sheet containing small holes. This was interposed between two more sheets on which anode and cathode electrodes had been deposited as row and column electrodes (Bitzer & Slottow 1966; Bitzer *et al.* 1966). The first panels used neon, but soon the standard design used UV emission from xenon to excite phosphors giving red, blue and green pixels.

**Figure 24.**
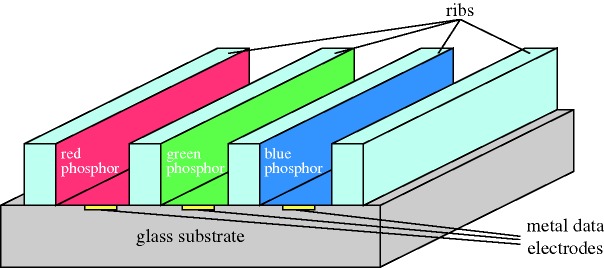
Schematic of phosphor arrangement in a plasma panel.

In modern panels the column electrodes are deposited as metal stripes on a glass substrate, and above them are a series of parallel ridges, dividing the display area into a number of narrow troughs. The sides of the troughs are coated with the phosphors (figure 24). The row electrodes are ITO stripes on the glass sheet that forms the top of the panel. The *I*–*V* characteristic of a gas discharge is an S-shaped negative resistance, and the working point is determined by the resistive load line. It is possible to energize the pixel by a certain voltage, and sustain it by a lower voltage, so often there are two sets of row electrodes. The driving voltage is 2–300 V.

Though the ridges reduce optical crosstalk, a fault of the original design, electrical charges on a pixel can influence neighbouring pixels in the same trough. This can be reduced by shaping the row electrodes, or, in the limit, by isolating each pixel cell. Nevertheless, it has proved very difficult to reduce the pixel dimensions below 0.5 mm, so that a high-resolution display smaller than 30 inch diameter is impracticable. On the other hand, very large displays are possible, and in the early period of flat-panel development, the large-panel market was dominated by plasma panels.

A brightness of 1000 cd m^−2^ was readily available, with excellent colour rendering, a wide viewing angle and a rapid response time. However, the efficiency of light production was low, so the power consumption was as high as for a CRT. Moreover, the need for high voltages made the electronics quite expensive. For some years plasma panels had a reputation for short life. The phosphors degraded, and the blue material was particularly bad. A life of 10 000 h was not uncommon, though manufacturers today would claim a much improved performance. One other fault has proved more difficult to overcome. This is the tendency for the display to hold the memory of a persistent bright image.

Commercial interest in plasma display panels (PDPs) was high from 1990 onwards, with first Fujitsu in the lead and then competition entering from many Far Eastern companies. By 2004 normal economics reduced those interested to Fujitsu–Hitachi, Pioneer, LG, Samsung and Panasonic, with the last two holding two-thirds of the market. In 2008 Panasonic claimed to have made the largest flat-panel display in the world, with a 150 inch diagonal (Ashida *et al.* 2008). The PDP market grew from 2000, as the lure of the CRT was fading, and the advantages of the LCD had yet to become apparent. The superb performance of the panels convinced many that the device had a promising future, but the manufacturing problems were not common knowledge. In fact, it was a difficult device to make in large quantity, and the cost of production was high. The emergence of rival display effects persuaded Pioneer and Fujitsu to abandon plasma panels early in 2009, and the fact that the total market is reducing may well mean that plasma panels will become a niche commodity.

#### (iv) Flat cathode ray tubes

The engineering world had been very conscious in the mid-1900s of the disadvantages of the CRT display, and it was not sitting idly by waiting for an alternative to appear. The main problem was the cumbersome shape, which was a great hindrance in moving to a larger screen size. The designers of a flat replacement had to face at least two severe problems. The first was to arrange the gun, itself quite long, within the depth of the tube, and then deflect the beam through two right angles without losing focus. The activation of the phosphors available at that time required almost 10 kV, and the beam current needed for a 40 inch screen would approach a milliampere. Such beams are difficult to deflect. The second problem is the vacuum within the tube. Safe practice demands that the thickness of the glass face plate must be at least 1/15 of the unsupported width, so that a 30 inch diagonal flat panel would need two plates over an inch thick and would weigh nearly 50 kg. One solution is to use supports bridging the gap, but these may interfere with the picture.

One prolific inventor who attempted to find a solution was Dennis Gabor, a Hungarian who first worked for Siemens in Germany, but came to the UK in 1934, and later earned a Nobel Prize for holography. His interest in CRTs was shown by a number of patents, including some on tubes that had ‘relatively small depth’, but had an elliptical cross section to accommodate the electron gun (Gabor 1955, 1956, 1957, 1959). He did not claim a truly flat tube until 1962 (Gabor 1962; Gabor *et al.* 1968).

The other early inventor was Ross Aiken, who started his research in 1951, but waited to file patents until he was sure he had the best design, and could get financial support for the development. He secured that support from Kaiser Aircraft in 1953 (Aiken 1984), but when he filed his patent in December (Aiken 1953), he learned that Gabor had anticipated him. There followed a patent battle, which was resolved with Aiken having the US rights, and Gabor the rights in the UK. Neither tube was ever produced on a commercial basis.

Ten years later RCA mounted a serious programme aimed at a 40 inch flat-panel colour CRT. Two approaches emerged. In that led by John Endriz, an x-array of 500 line cathodes fed electrons into 480 vanes of secondary emission multipliers (Cosentino *et al.* 1979; Endriz *et al.* 1979). The other team, including Tom Credelle and Tom Stanley, favoured a normal thermionic cathode injecting electrons into a series of box guides at 90° to the line of the gun. These boxes, which acted as supports between the two display faces, contained a series of plates to deflect the electrons through a further 90° on to the phosphor (Stanley 1975, 1977; Siekanowicz *et al.* 1976; Credelle 1977).

Other attempts were made by Texas Instruments (Scott 1977) and Siemens (Graefelfing *et al.* 1968), but by 2000 it was generally appreciated that the problems of brightness, resolution and display size could not be met in displays that would be competitive commercially. Any solution would have to come from a design that bore little resemblance to the CRT. No matter how clever inventors were in twisting and bending electron beams, the obvious way forward was to make a cathode that was itself a plane, and that could be arranged parallel to the phosphor screen. It would be difficult to do this with a thermionic cathode array, since the screen would itself get hot, not a desirable attribute.

**Figure 25.**
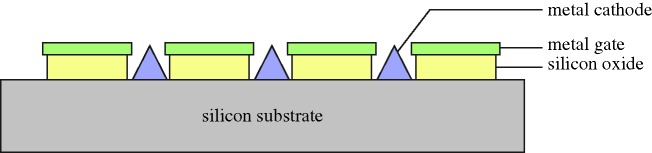
Schematic of the Spindt cathode.

Ken Shoulders and Louis Heynick, working at the Stanford Research Institute (SRI), were the first to appreciate that the answer could come from cold cathodes, with the field required to extract electrons reduced by shaping the cathode into a point. They made small cathodes as a clump of needles of molybdenum or tungsten, formed by coating the surface with aluminium, and then heating until the Al melted (Shoulders & Heynick 1966*a*,*b*). They observed that surface tension then caused material to migrate into the needle shapes, but candidly admitted that they did not understand the mechanism. A few years later Capp Spindt collaborated with Heynick to invent a more practicable manufacturing method (Spindt & Heynick 1970). They evaporated Mo on to a plane surface, and used an ingenious shadowing technique to form minute cones (figure 25).

Their success caused many others to join in the search for a flat CRT, and the Spindt cathode became the standard basis on which flat CRTs were developed (Spindt *et al.* 1991). A number of companies were formed to make devices, but their success was limited, and most saw a period of serious investment before it was realized that the technical problems militated against commercial success. Monochrome tubes were made, in sizes up to 12 inch, and colour tubes were described as producing a picture equal to that of a normal CRT. However, it was difficult to get sufficient sustained emission for a screen brightness above 500 cd m^−2^, and production costs were high. The major player was Candover, effectively a spin-off from SRI founded in 1990, which attracted funding of over $300 million. In 2004 they sold their technology to Canon for $10 million, and abandoned the field. PixTech, a French spin-off from LETI in Grenoble, with a US subsidiary, was founded in 1992, and they also tried to market displays using their own version of Spindt cathodes. They had a limited success, and demonstrated 15 inch colour tubes at exhibitions in 1999, but there has been little activity in the last five years.

**Figure 26.**
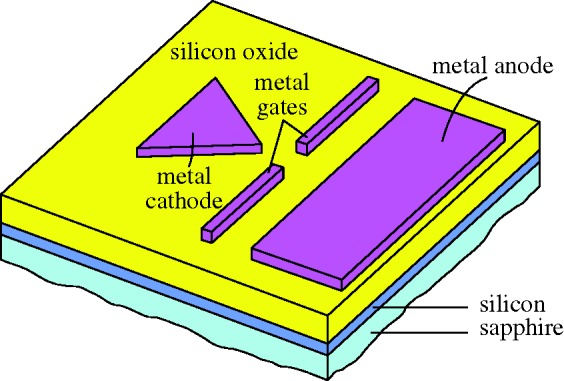
Schematic of the GEC planar cathode.

Among the companies interested in cold cathodes was the UK General Electric Company (GEC). Their main potential application was microwave tubes, but they were conscious of the display possibilities. A group at the Hirst Research Centre led by Rosemary Lee first concentrated on making microtips from silicon, since they thought that the production techniques were simpler than those for Spindt cathodes. They did succeed in making efficient cathodes that were stable (Lee *et al.* 1989), but it was clear that this technique could not give cheap large-area sources. However, in 1987 the group also invented a planar version, which was potentially suitable for large-area metal cathodes (Lee & Cade 1987, 1988; figure 26).

There was clear merit in a surface structure that attracted electrons parallel to the surface and scattered a proportion of those incident on the anode towards a second anode on the other side of the panel. The surface emitting display (SED) could be fabricated by the techniques perfected for chips, albeit on a larger scale. Many variants of the planar cathode were invented subsequently, with the lead taken by Canon and Futaba. Futaba, with a history of manufacturing small vacuum fluorescent displays, showed that carbon nanotubes deposited on the surface gave a larger and more stable current than metal edges (Itoh *et al.* 2007). Canon also made steady progress. They teamed first with Toshiba, and, after this collaboration ended because of a legal dispute with Applied Nanotech, with Hitachi and Matsushita. At regular intervals after 1998 it was announced that commercial production was imminent, with the press releases after 2006 given weight by the impressive panels shown at exhibitions. In 2006 Canon demonstrated a panel with a brightness of 450 cd m^−2^, a contrast ratio of 50 000 : 1 and a response time less than 1 ms. However, 3 years later there is still no sign of commercial production of TV panels. Obviously the SED is not easy and cheap to make.

## 5. Components for addressing

In §2 I discussed the need for an electrical nonlinear component to be integrated on the panel. This need came to the fore in the 1970s, when the potential of LCD panels was becoming apparent, but there was no candidate component for addressing. It must be admitted that this caught the international semiconductor expertise off balance, in that almost all effort had been directed at growing large perfect single crystals, and then depositing thin layers of controlled purity epitaxially on the crystal slices. Materials experts felt that glass substrates were incompatible with silicon, germanium and the III–V compounds, and those semiconducting compounds that could be deposited on glass, like cadmium selenide and zinc sulphide, would never give stable performance for the several years that companies expected. Instead they turned to metal–insulator–metal sandwich diodes, with combinations like Ta/Ta_2_O_5_/Cr, or glass switches, made of combinations incorporating chalcogenides, such as GeAsTe (Ovshinsky & Fritzsche 1973; Adler *et al.* 1980). Though some of the research on these components was generously funded, and enjoyed collaboration with internationally renowned physicists, stability, reproducibility and long life were never obtained.

The solution came from the semiconductor workhorse, silicon, but in an unusual form. In the 1960s, a group led by Henry Sterling at the Standard Telecommunications Laboratory at Harlow had shown that the glassy form of Si, normally considered an insulator, could be made partially conducting if thin films were deposited in a hydrogen atmosphere (Chittick *et al.* 1969). The research was continued at Dundee University by Walter Spear and Peter LeComber, and in 1975 they showed that the conductivity could be controlled by the addition of the same impurities as were used for doping crystalline Si (Spear & LeComber 1975). This discovery inspired worldwide efforts that led to the first practical solar cells. At that time, the interest in renewal energy was beginning to gain momentum, and it was not surprising that Spear saw that his priority should be a focused strategy aimed at keeping his research group at the head of the international a-Si solar cell effort, leading in the basic physics.

**Figure 27.**
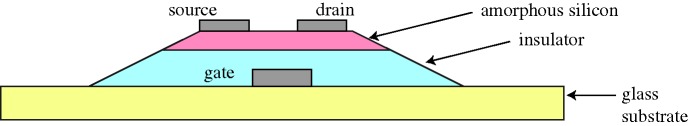
The first amorphous silicon thin-film transistor.

**Figure 28.**
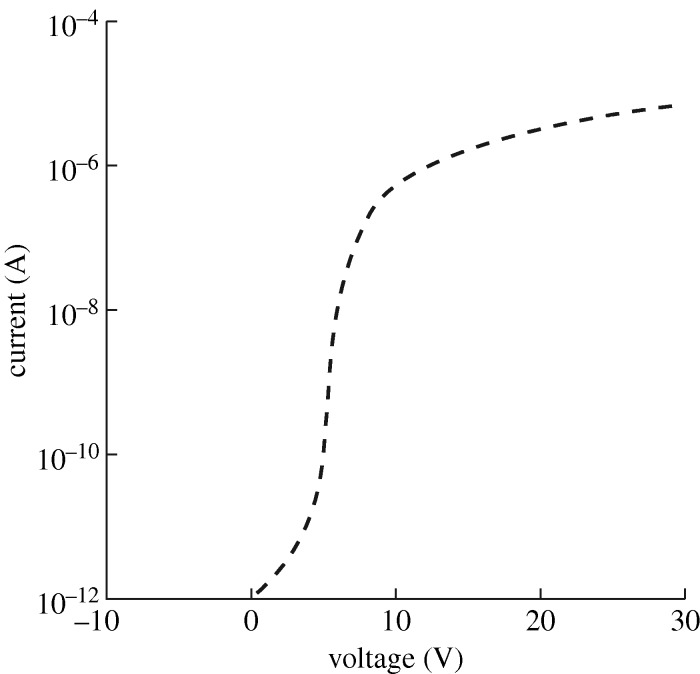
Electrical characteristics of the first a-Si TFT, made at Dundee University.

The Displays Group at the Royal Signals and Radar Establishment (RSRE), Malvern, had a different view. They saw a UK laboratory in possession of the key to addressing displays. They were sure, following instinct rather than science, that a-Si TFTs would work with LCDs, but they lacked the facilities for making the devices, no easy task. Persuading Dundee to collaborate, while their eye was on a different ball, took many months. In any case, Spear had reservations about collaboration with non-academic organizations (Adams 2009). It was LeComber, without Spear’s blessing, and possibly without his knowledge, who eventually made the original a-Si TFTs, and supplied the first batch to Malvern. RSRE switched LCD pixels with them on Xmas Eve, 1978. The structure of the TFT and its characteristic are shown in figures 27 and 28 (P. G. LeComber 1978, personal communication). Later work at Dundee and Malvern confirmed the potential of a-Si for displays (LeComber *et al.* 1979; Snell *et al.* 1981).

Neither group was able to gain financially from this development, because the original Weimer patent covered the TFT as a device, and did not specify any particular semiconductor. The two groups consulted patent experts, but were advised that an attempt to license the invention would certainly involve legal action, which they were unlikely to win. So this discovery, which is still widely used worldwide, brought no direct economic benefit to the UK.

Though the basis for progress was now established, there were many hills to climb before industry was convinced of the virtues of this new approach. One major advantage was the use of silicon, for industry knew that this semiconductor was stable, unlike the sulphides and selenides previously on offer. This conviction was not severely undermined by reports of changes in solar cell performance during prolonged exposure to sunlight. The cause of this effect, first reported by Staebler & Wronski (1977), is still obscure, and it was not highlighted during the early days of a-Si addressing. Indeed, the protagonists of more exotic materials like CdSe for TFTs could well have argued that the playing field was not level, for they were criticized for changes in device properties during life that were no worse than those for a-Si (Weimer 1962*a*; Lee *et al.* 1992; Brody 1996). However, fair play was never a feature of displays commercialization, and it is true that much research and huge funding ensured that stability was eventually obtained, at least in a-Si arrays for LCDs. Nevertheless, it was many years after the first pixel was switched at Malvern before large arrays could be produced in industry with a high yield.

Amorphous silicon has proved essential in the progress of flat-panel displays, but its semiconductor properties are undistinguished. In particular, the field effect mobility is rarely as large as 1 cm^2^ V^−1^ s^−1^, a thousand times lower than that of crystalline Si. This means that the material can be used only for the addressing switches. The display requires drivers and data control circuitry as well, and if a-Si is used for the switching, standard chips have to be fixed to the panel. A dream of the early workers was that a semiconductor would be found that would have a high enough mobility to accomplish all the necessary operations. The mobility needed was at least 10 cm^2^ V^−1^ s^−1^.

The answer came from standard chip production, where polycrystalline Si layers were routinely deposited by chemical vapour deposition (CVD) of silane, though the deposition temperatures were well above the softening point of glass. However, it was discovered by Doug Meakin and Piero Migliorato at GEC, Wembley, in collaboration with Nico Economou of the University of Thessaloniki, that lowering the chamber pressure from the standard 26.6 Pa to below 1.3 Pa allowed growth at under 600°C of polycrystalline Si films with a mobility of 10 cm^2^ V^−1^ s^−1^ (Meakin *et al.* 1987). This triggered other laboratories to work on poly-Si, with an emphasis on alternative production methods. The favoured method was laser annealing of a-Si, and mobilities gradually increased. The leading companies were, and still are, Seiko–Epson (Jiroku *et al.* 2004) and Sony (Sekiya *et al.* 1994), and both have reported poly-Si TFTs with mobilities of several hundred cm^2^ V^−1^ s^−1^. Seiko was using the TFTs for integrated drivers. The two companies may well combine their display activities soon.

Though the electrical properties of poly-Si are surprisingly good for a film containing many grain boundaries, the production process is costly, and has not been used for large displays. Alternatives are being sought. Research has been reported on several semiconducting oxides, including zinc oxide and indium oxide, each of which can have field mobilities much greater than that of a-Si (Levy *et al.* 2008; Park *et al.* 2008; Wang *et al.* 2008). In addition, progress on semiconducting organics for LEDs has led to interest in fully organic back planes, and a number of organic TFTs have been reported. CDT, using 6,13-bis(triisopropylsilylethynyl) (TIPS) pentacene, has made TFTs with a gate width of 50 μm and mobility up to 1.8 cm^2^ V^−1^ s^−1^, with little reduction for gates as narrow as 5 *μ*m, demonstrating the low contact resistance, a key point in organic TFT research. CDT has also obtained, using a proprietary material from Merck, a TFT with mobility above 3 cm^2^ V^−1^ s^−1^ (J. H. Burroughes 2009, personal communication).

Though early work aimed at replacing a-Si TFTs, and used glass substrates, more recently there has been emphasis on solution or ink-jet printing, and deposition on plastic substrates for flexible displays. An early leader was Plastic Logic, spun out from the Cavendish Laboratory, which has made TFTs by ink-jet printing on plastic substrates with mobilities approaching 1 cm^2^ V^−1^ s^−1^. Another forward step has been the formulation by CDT, in collaboration with Silvaco Data Systems, of an equivalent to the SPICE simulator for electronic device automation. There is little information on the life of organic or polymer TFTs, though it would be surprising if this were as good as for a-Si or poly-Si, particularly for devices on plastic substrates.

## 6. Applications

The spectrum of modern uses of flat-panel displays ranges in size from microdisplays, requiring optical assistance to view them, to the huge displays now common in sports stadia, and it encompasses many types of embodiment. Though there may well be significant differences in the types of visual effect used for some applications, I will here concentrate on a few applications that demonstrate the breadth of the technology.

It should not be forgotten that even a simple flat panel may yield a considerable advantage in cost and complexity over alternative ways of presenting data. For example, long before active matrix techniques were available, automobile manufacturers were considering incorporation of LCDs or EL panels into the driver’s display. Not only was the thickness of the display reduced from 60 to 12 mm, but the number of parts and processes came down from 430 to 35.

### (a) TV panels

**Figure 29.**
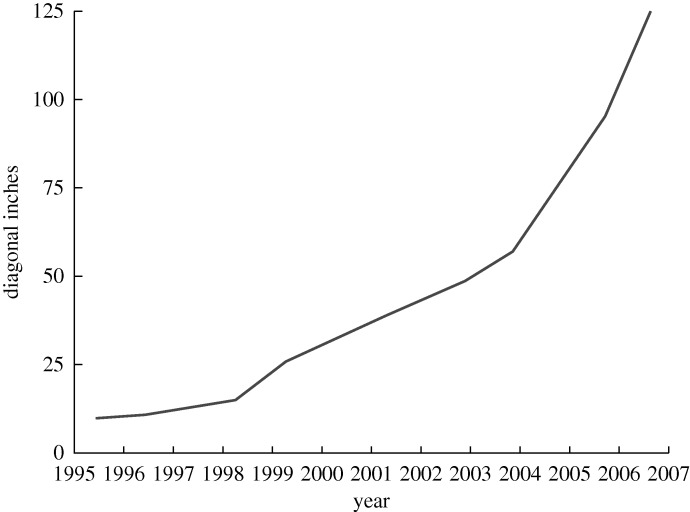
The growth in manufacturing capability of active-matrix LCD panels.

There were two trends in the consumer market after 2000 that resulted in the demise of CRT TV sets. The first was the realization by computer users that a flat panel gave them extra desk top space. They were willing to pay a considerable premium in price for this privilege. The second was the desire of households worldwide to view a bigger TV picture. There was absolutely no fault with the CRT picture—it was bright, sharp, could be viewed at all angles, and was inexpensive. Certainly the brightness and colour purity faded with time, but many households were happy to keep the same set for ten years or more. The change happened very rapidly. It was, of course, made possible by the improved skills of the manufacturers, which learned how to make a-Si TFTs with high yield over large areas, illustrated in figure 29.

The results on sales were surprising. In the UK, Dixons, then the largest retailers, reported that in 2004 nearly 90 per cent of the TV sets sold were CRTs. In 2005 the percentage had dropped to 20 per cent, and in 2006 had dropped to 5 per cent. Dixons stopped selling CRT TVs the next year, and were shortly followed by other retailers. However, CRTs still retain niche markets, particularly when high resolution is required.

**Figure 30.**
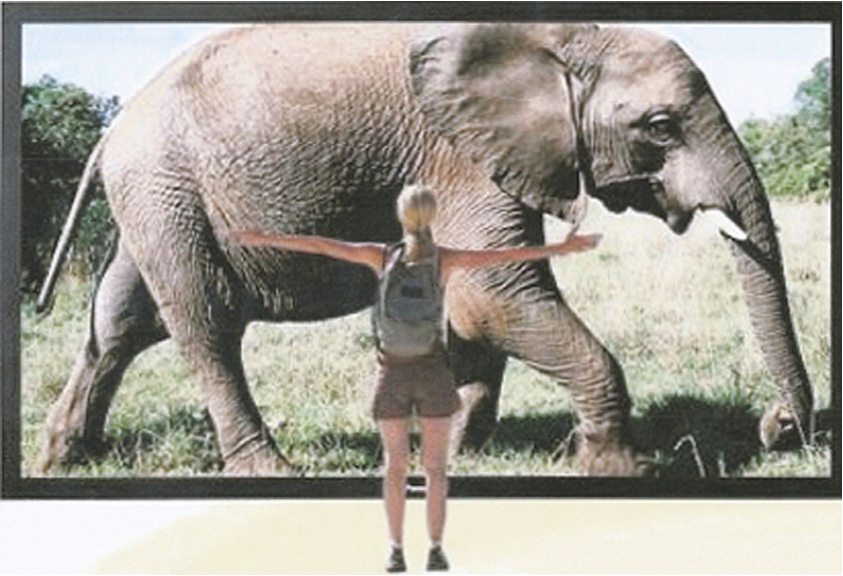
A 150 inch Panasonic plasma panel TV.

The move towards larger TVs had benefited plasma panels (PPs), which were previously reserved for specialist applications, but after 2000 they dominated the flat-panel TV market for screens larger than 40 inch. By 2006 the available PP size was just over 100 inch, though larger panels had been demonstrated (figure 30). Very large LCs were, however, also becoming available.

**Figure 31.**
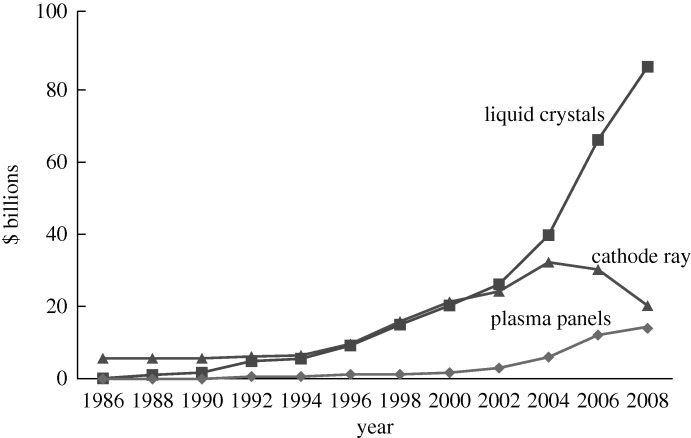
World sales of flat panels, 1986–2008.

The two technologies now compete aggressively in the market for large TVs, with each having protagonists in their views on the excellence of the picture. PPs certainly had an early edge here, but LCD manufacturers have made many improvements in back lighting, power consumption and angle of view, so that there is now little to choose between them. The current market position is shown in figure 31.

**Figure 32.**
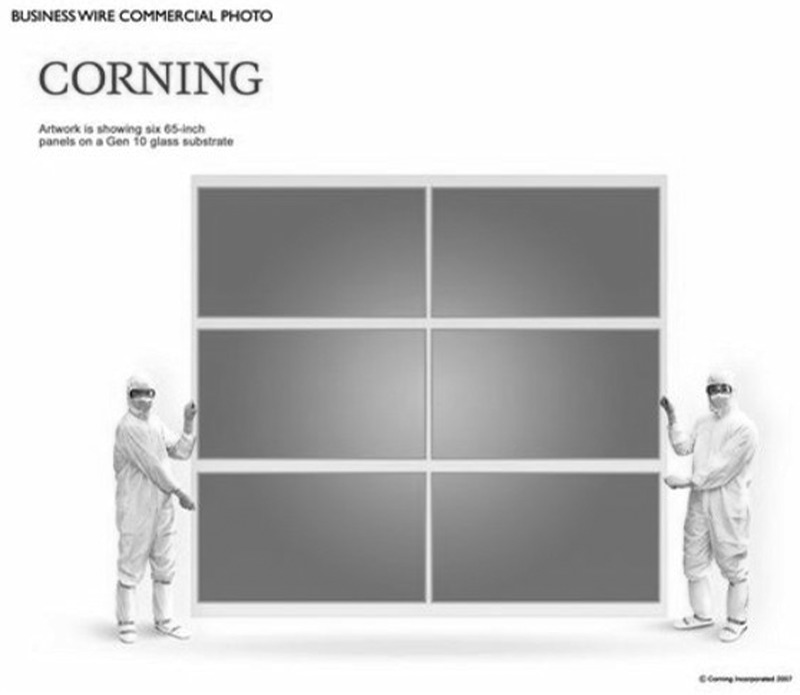
Generation 10 glass for displays.

PPs will face great pressure from LCDs and OLEDs in the future. LCD manufacturers are investing heavily in increasing capacity and reducing costs by standardizing on huge glass areas, and the sheet of glass from which displays are cut is now Generation 10, 2880×3130 mm (figure 32). Corning plans to invest over $400 million in a plant for Sharp at Sakai City. Samsung does not intend to lag. Their plans are for a plant using Generation 11 glass, 3000×3320 mm, which would give six 72 inch LC panels from one sheet.

The outlook for OLEDs in TV is not certain. Though impressive OLED panels have been shown in exhibitions for a number of years, the UK consumer had to be patient until early in 2009 before they could buy one. Sony then announced a screen 11 inch wide with excellent picture quality. The price, however, illustrated the manufacturing problems. The first announcement quoted a price near £3500, though this was quickly followed by offers to import panels from the USA at half that price.

It is seductive to present any light-emitting technology as simpler than, for example, an LCD, because no additional source of illumination is necessary. The counter argument is that LCDs separate the problems, so they can all be attacked individually. The progress in LCD picture quality attests to the validity of that argument, for much of the recent research has concentrated on back lights, angle of view, power consumption economies and other factors that have had lower priority while the technology achieved its leading market share.

Many of the OLED production problems arise from the interdependence of many of the display’s functions. The panel must be able to show the three primary colours, and the red and the green emitters are eminently satisfactory. The blue, on the other hand, has too short a life. This means that the colour balance of the picture will change during its use. One method of preserving colour balance is to use red, green and blue phosphors excited by a deep blue or UV source, but here it is that source which is short-lived.

Another problem is the addressing. The separation of functions in the LCD means that the a-Si TFTs are just switches, and they carry a minimum current. In OLED panels, the current energizing the emitters has to pass through the TFTs, and they therefore are driven hard throughout their life. Life changes due to trapping effects in a-Si are of minimal importance in LCDs, but not in OLEDs. As a result, a number of OLED manufacturers have turned to poly-Si, which is more stable, but more difficult to make. In addition, the need to pass current to pixels in the middle of the panel throws an additional load on the quality of the transparent conductor. It is not then surprising that the heralded arrival of OLED TV has been delayed, though the penetration into less demanding markets has continued apace.

### (b) Flexible displays

In the early days of flat panels, there was some concern about the glass substrates. The risk of breakage was, perhaps, emphasized, and there were some experiments with plastic sheets. In time, however, glass became the standard, and there it has remained. However, at some stage defence users again became conscious of their dependence on an admittedly fragile component, and began funding research on plastic substrates. They took this initiative further, asking for a display that was flexible, with a vision of field maps that were portable, and could be rolled up. In fact, a form of flexible display had been available to the consumer for many years, a temperature indicator containing cholesteric LC. These were marketed widely, and since they were cheap, the customer rarely complained when their ability to read the temperature lapsed after some months. That, however, was symptomatic of the problem of using plastics. Their ability as a moisture barrier is far less satisfactory than that of glass, whose main weakness in that regard is the adhesive seal between the two sheets. The requirement for a display substrate is a water vapour transmittance less than 10^−6^ g m^−2^ d^−1^ and an oxygen transmittance less than 10^−5^ ml m^−2^ d^−1^, whereas the best common plastics, PET and PEN, are several orders of magnitude worse than this.

The demand for moisture and oxygen barrier coatings has attracted much research activity, for the potential applications are broader than just flexible displays. Included in these were solar cells, which obviously have to be exposed continuously to the weather. Many companies have undertaken research, including 3M, Vitex, Dupont and Batelle, and many government and university laboratories have also been involved.

The general presumption is that no single material will meet the harsh specification, so most recipes involve a series of organic–inorganic layers, sometimes with a thin metal film as the inorganic layer. Diamond, hard carbon, has been proposed by a number of research workers. The actual measurement of moisture penetration is not easy. The standard technique has a limit of 0.005 g m^−2^ d^−1^, well above the requirement. As a result it is difficult to assess whether the films available commercially are good enough, since they usually just quote performance as better than the standard limit. Early in 2008, the Singapore Institute of Materials Research and Engineering (IMRE) announced that they had developed a film that was 1000 times more effective than any technology on the market. This had some credibility, since IMRE had previously invented test sensors that claimed to be capable of measuring water vapour permeability rates of 10^−7^ g m^−2^ d^−1^. The claim was that incorporating Al_2_O_3_ nanoparticulate matter into a stack of layers of acrylic polymer, ITO, Al_2_O_3_ and ITO on a polycarbonate film gave very low permeation, and a series of three stacks met the device requirements. Titanium nanoparticles could be used together with or instead of aluminium oxide (Ramadas & Chua 2006). Nevertheless, over a year later there has been no journal publication, and no independent verification, so judgement must be deferred.

It seems unlikely that all the production and life problems of making large flexible video displays will be solved swiftly. Smaller displays, which give more freedom to the instrument designer because the display can be conformable, should be available in the near future.

### (c) Electronic paper and electronic books

An electronic book, now generally known as an E-book or Reader, is the digital equivalent of a printed book, in the simplest versions capable of presenting monochrome text one page at a time, with page changing taking a second or two. One of the first people to try to capitalize on the advantages of an electronic book was Robert Maxwell, who approached the author at RSRE, Malvern, in 1981, with a project to make a portable LCD capable of reproducing a page of text. Maxwell was then Chairman of the British Printing and Publishing Corporation, which owned several UK national newspapers, but his interest stemmed from his ownership of Pergamon Press Ltd, which published many legal and medical tomes. These were expensive to produce, and new editions were needed every year. He thought that an electronic memory and display was the way forward, and was prepared to finance generously an R&D project. He stated that money was no real object. The concept was clearly ahead of its time, since active matrix techniques were still in their infancy, and adequate data storage was not then available within the dimensions of a textbook. The project had to be deferred, probably fortunately, in view of his subsequent history, but the concept was certainly valid.

It is simplest to think of an E-book as a Reader in which the visual effect is provided by a flat or flexible electronic display that rivals the effect of print on paper, and so is called electronic paper. A number of versions of the E-book are now on the market. The input data come from inbuilt memory, or via a wireless connection. The screen size in early versions approximates to a paperback book, though recent models are as large as A4 paper. Some designs are based on plastic films, largely to reduce the weight and make the device robust. One important feature of the E-book is its portability, so that the power must be supplied by a rechargeable battery. This militates against emissive visual effects, though back lights can help for viewing in poor light. There is obviously a premium on high reflectivity and high contrast.

The type of electronic paper that is most widely used exploits electrophoresis (EP). This effect was discussed previously, in §4*a*(ii), and there it was explained that the early technology was found wanting because the pigment particles clumped, settled under gravity, and left stains on the electrodes. Modern EP cells reduce these problems by either microencapsulating the pigment particles in tiny spheres within the carrier fluid, or by dividing the panel volume into segments by barriers between the surfaces.

**Figure 33.**
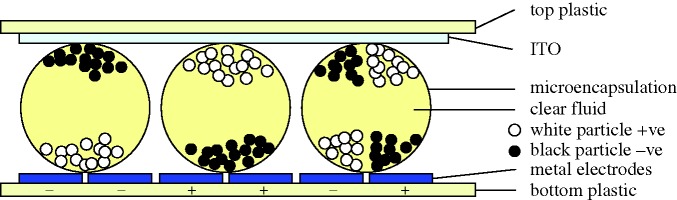
Schematic of structure of E Ink paper.

The pioneering research on microencapsulation was done by E Ink, which collaborated with research scientists at MIT (Jacobson *et al.* 1997; Comiskey *et al.* 1998). They also worked closely with Philips, at Heerlen and Kobe, and the structure they devised is shown in figure 33 (Henzen *et al.* 2004). The main differences from the classic form shown in figure 14 are the encapsulation and the use of two sets of pigmented particles, one set black and negatively charged, the other white and positive. The fluid within the sphere is clear. In principle, the positive particles could be coloured, or there could be micrometre-sized colour filters on the front surface. Commercial e-paper has a reflectivity of 40 per cent and a contrast ratio of 7 : 1. White paper has a reflectivity of 85 per cent.

Operation of an EP display requires long positive and negative pulses, and this complicates addressing. The response time is set by the transit time of the pigment particles, and for E Ink paper this is just less than a second a page if grey scales are to be shown. The paper is used in the Sony Reader and the Kindle, which both use glass substrates. There has been some concern about the use of glass substrates in the larger Readers, which could be A4 size. A flexible Reader using E Ink, which is planned to reach the market early in 2010, is made by Plastic Logic in Dresden. This incorporates polymer TFTs (Burns *et al.* 2006).

Production of a rival EP display was announced by Bridgestone in April 2009. The medium is a ‘liquid powder’, and this is held between two plastic plates, with barriers between the plates to prevent pigment motion (Sakurai *et al.* 2006). Small colour displays have been used for supermarket labels, and now displays of A4 size are coming on to the market.

Though constraining the volume available to particle diffusion in EP displays can certainly reduce clumping and gravitational settling, it does not eliminate them, and electrode staining, with a higher voltage needed to remove particles than in first use, is still a problem. There is little published on the life of electronic paper based on EP, but the patent literature shows that the reflectivity of early models halved within 2000 h. Modern paper will show some improvement on that figure.

**Figure 34.**
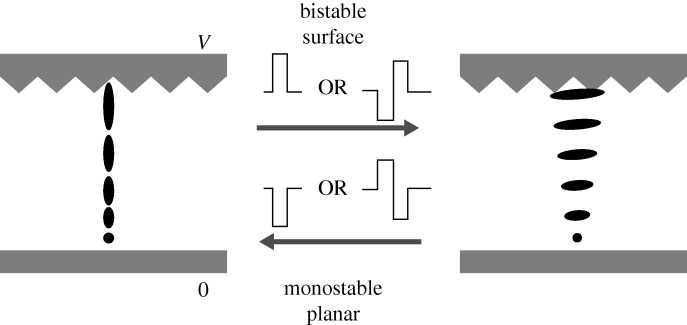
Bistable LC states in ZBD display.

**Figure 35.**
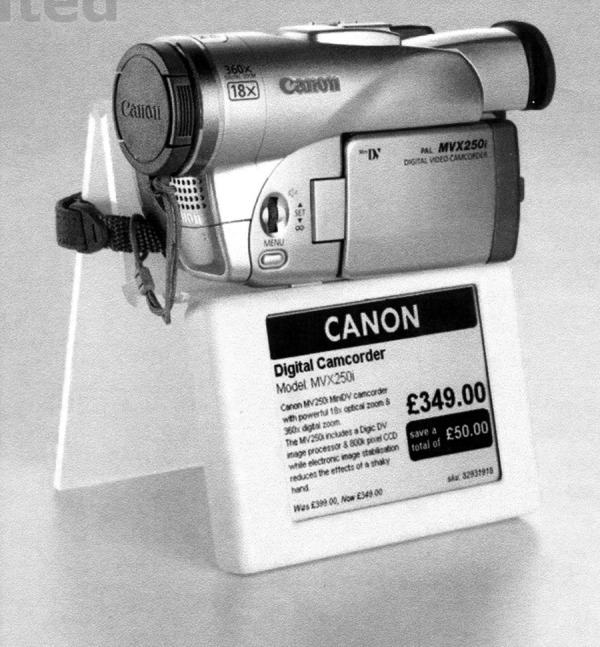
A ZBD shelf label display.

One requirement of visual effects for electronic paper is bistability, since power is then required only when the display needs updating. If the bistable states are separated by a significant energy barrier, so that transition requires a short pulse with voltage above a threshold, passive matrix addressing is possible, with much simplification in back plane design, and a considerable reduction in processing cost. Though standard TN LCDs are not bistable, a change in the design can give this property. ZBD Displays, following earlier research on LC grating alignment (Flanders *et al.* 1978), adds a grating structure to one surface of a TN display, and there are then two stable states (figure 34). Switching from one state to the other requires a short pulse, so the updating of a small page requires only 250 ms. The display has a reflectivity of 39 per cent and a contrast ratio of 10 : 1, surprisingly good figures considering the need for polarizers (Jones 2007). The main use of the technology is for shelf-edge labelling (figure 35), but there seems no technical obstacle to an increase in size.

Nemoptics obtains a similar effect without the grating. They treat one surface so that the anchoring of the LC molecules is weak, and obtain two stable states with the nematic molecules parallel to the surfaces, one twisted and one aligned. Molecules can move from one state to the other only by passing through a third state, with molecules orthogonal to the surfaces. This transition is made by unipolar pulses of different shapes for writing and erasing (Faget *et al.* 2006; Dozov *et al.* 2007). The display reflectivity is 33 per cent, with a contrast ratio of over 10 : 1. An A4 page can be refreshed on 0.4 s. Nemoptics displays are available in colour, under the trade name BiNem, and are being used for shelf labelling. In November 2007 they announced that Seiko Instruments would be using BiNem for a Reader.

Though both ZBD and Nemoptics have made experimental displays on plastic substrates, there is no information on life. The glass versions have the life of normal LCDs, and, where weight and flexibility are not a prerequisite, could provide an E-book with a simpler back plane, presumably at a lower cost than the electrophoretic versions.

This is a dynamic field, and while EP has produced commercial devices, and bistable TN has demonstrated small working prototypes, there are many reports of visual effects that are claimed to have superior properties and immense potential for E-books. Proposed effects include electrowetting, electrofluidics and bistability in cholesteric LCs. Many mechanical effects in microelectromechanical systems (MEMS) are also under consideration.

### (d) Other applications

**Figure 36.**
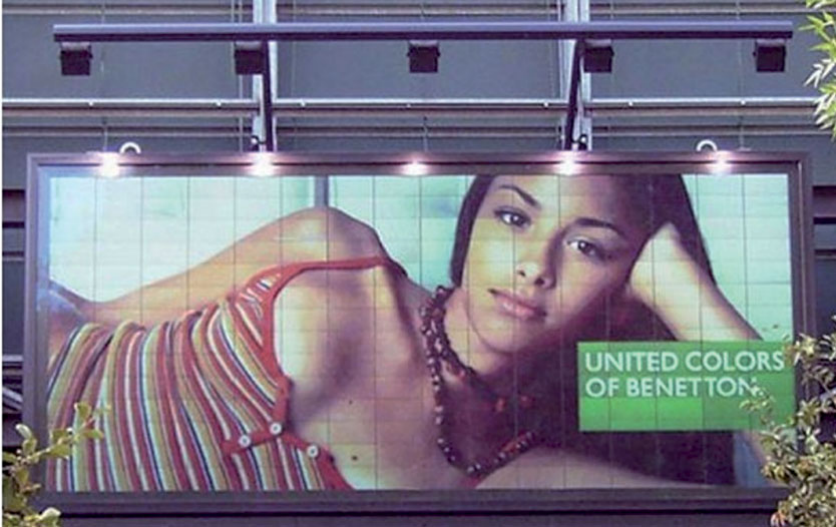
Large cholesteric liquid crystal billboard (courtesy Magink).

There are a number of additional applications of flat-panel electronic displays, but though the uses differ greatly from those covered earlier, the physics and chemistry are similar. The exception is the very large displays, such as those used for video presentation in sports stadia. They use several million LEDs, generally in tiles about a metre square. Smaller displays used for signs and billboards can exploit cholesteric LCDs. The sign shown in figure 36 is constructed from over 200 tiles to give an area of over 10 m^2^, with a reflectivity of 32 per cent. The picture can change in 1–2 s (Coates 2008).

Any flat-panel display lacks reality unless it can show three dimensions. The simplest method requires the user to wear polarized spectacles, a disadvantage for many users. Most other attempts give a good stereoscopic image to one observer, but others can see a disturbing pseudoscopic presentation. The most convincing representation comes from computer-generated holography, but this involves much computation, and is very expensive.

## 7. Future trends

Market predictions are always suspect, because they assume that consumer behaviour is like ‘the law of the Medes and Persians, which altereth not’ (*King James Bible*, Daniel 6:8). Predictions based on technological progress are even more questionable. Nevertheless, a survey of flat-panel history and achievement would be incomplete without some words on the future. Some developments seem clear. The TV market will see competition between LCDs and the various forms of OLED, with plasma panels decreasing in importance. The conventional CRT will die away, and it is unlikely that any flat form will become significant.

Competition in smaller devices is more difficult to assess. Flexibility can be an important feature in design, but its advantages pale if linked with a short display life. Nevertheless, if a fashionable item is inexpensive, its life is peripheral. That would indicate an opening for flexible OLEDs in mobile phones, but problems in Readers. The limited success to date of the research on barrier layers gives concern, because much effort and funds have been applied. If the reports of success from Singapore remain unconfirmed, glass Readers would prevail, with a possible opening for passive matrix bistable LCDs.

Silicon is truly a workhorse in electronics, and its versatility has been shown in the Texas Instruments Digital Light Projector. The technology has a natural home in microdisplays, and it is certainly possible that interesting optical effects can be obtained by sub-micrometre motion of layers in MEMS. Ingenuity will be needed, however, before the high definition possible in small chips can be projected to larger areas within the limited depth of Readers.

A future without three-dimensional TV displays is unimaginable, and a few companies have made experimental models that come close to a satisfactory presentation. The interim steps of the Dolby three-dimensional Digital Cinema, using polarizing goggles, and the Planar Stereo Monitor, involving two screens, may well kindle consumer interest enough for companies to market displays that approximate to reality in a form acceptable to consumers.

The past 40 years have seen immense progress in flat-panel technology, and has led to huge funds being applied in the many areas where panels can provide excellent images. The future is likely to be as bright for displays as the past has been rewarding for physicists, chemists and engineers.

## Footnotes

1 In the UK, English Electric Valve Co., Chelmsford, were licensees.

2 Many of the events that follow are not referenced, because they have never been published. They are based on the recollections of the author, who from 1967 to 1983 led the Displays Group at RRE and was responsible for the UK Government-supported Civil and Defence Displays programmes.

3 A fuller version of some of these events is given in Hilsum (1984).

4 Martin Pope’s lifelong contributions to organic electronics are set out in http://chemxserver.chem.nyu.edu/MPope/scientific%20History.htm.
